# CRL4^Wdr70^ regulates H2B monoubiquitination and facilitates Exo1-dependent resection

**DOI:** 10.1038/ncomms11364

**Published:** 2016-04-21

**Authors:** Ming Zeng, Laifeng Ren, Ken'Ichi Mizuno, Konstantinos Nestoras, Haibin Wang, Zizhi Tang, Liandi Guo, Daochun Kong, Qiwen Hu, Qun He, Lilin Du, Antony M. Carr, Cong Liu

**Affiliations:** 1 Key Laboratory of Birth Defects and Related Diseases of Women and Children (Ministry of Education), Department of Paediatrics, West China Second University Hospital, Sichuan University, Chengdu 610041, China; 2 Department of Medical Microbiology and Immunology, Fenyang College of Shanxi Medical University, Fenyang 032200, China; 3 Genome Damage and Stability Centre, School of Life Sciences, University of Sussex, Brighton BN1 9RQ, UK; 4 National Laboratory of Protein Engineering and Plant Genetic Engineering, College of Life Sciences, Peking University, Beijing 100871, China; 5 Department of Microbiology, College of Basic Medical Sciences, Third Military Medical University, Chongqing 400038, China; 6 State Key Laboratory of Agrobiotechnology, College of Biological Sciences, China Agricultural University, Beijing 100083, China; 7 National Institute of Biological Sciences, 7 Science Park Road, Zhongguancun Life Science Park, Beijing 102206, China

## Abstract

Double-strand breaks repaired by homologous recombination (HR) are first resected to form single-stranded DNA, which binds replication protein A (RPA). RPA attracts mediators that load the Rad51 filament to promote strand invasion, the defining feature of HR. How the resection machinery navigates nucleosome-packaged DNA is poorly understood. Here we report that in *Schizosaccharomyces pombe* a conserved DDB1-CUL4-associated factor (DCAF), Wdr70, is recruited to DSBs as part of the Cullin4-DDB1 ubiquitin ligase (CRL4^Wdr70^) and stimulates distal H2B lysine 119 mono-ubiquitination (uH2B). Wdr70 deletion, or uH2B loss, results in increased loading of the checkpoint adaptor and resection inhibitor Crb2^53BP1^, decreased Exo1 association and delayed resection. Wdr70 is dispensable for resection upon Crb2^53BP1^ loss, or when the Set9 methyltransferase that creates docking sites for Crb2 is deleted. Finally, we establish that this histone regulatory cascade similarly controls DSB resection in human cells.

## Abstract (web summary)

The repair of double-strand DNA breaks by homologous recombination requires resection of the DNA ends. Here the authors show that in Schizosaccharomyces pombe and human cells, Wdr70 is recruited as part of the CRL4 complex to promote ubiquitination of H2B and allow Exo1-mediated resection.

## Graphical abstract

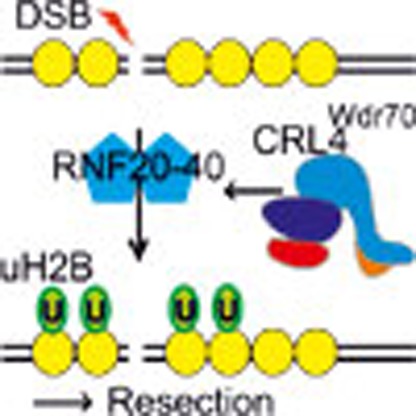

DNA damage, if not accurately repaired, poses a threat to genome integrity. When incorrectly repaired, a single DNA double-strand break (DSB) can result in loss of genetic information and gross chromosomal rearrangement, both signatures of carcinogenesis^1^. In metazoans, DSBs are mainly repaired by non-homologous end joining (NHEJ) or homologous recombination (HR). NHEJ requires minimal DNA end processing and is innately error prone because chemically damaged or lost bases are not replaced. In contrast, HR requires extensive 5′-to-3′ DNA end resection. This generates a stretch of RPA-coated single-stranded DNA (ssDNA) that attracts repair factors to build a Rad51 filament to initiate a homology search. Once a homologous template is found, repair is accurate because DNA synthesis can replace bases that were either lost or chemically modified at the original damage site^2^.

Resection is initiated by an Mre11–Rad50–Nbs1 complex (MRN) and the associated C-terminal-binding protein interacting protein (CtIP) repair factor^3^,^4^. DNA is nicked by the Mre11 nuclease on the strand with the 5′ end, ∼15–20 bases 3′ from the break. In the yeast model system, this Mre11 activity is regulated by the CtIP homologue^5^. The small region from the nick site to the break is subsequently degraded 3′ to 5′ back to the break, releasing DSB-bound proteins such as the NHEJ initiator Ku. MRN- and CtIP-dependent ‘clipping' provides an entry point for the 5′ to 3′ resection factors. Resection depends on either Exo1 or Dna2 (which function in complex with BLM: known as Sgs1 or Rqh1 in *S. cerevisiae* and *S. pombe*, respectively)^5^. Once resection is initiated, repair of the DSB is channelled into HR pathways such as sister chromatid repair or single-strand annealing^2^. Thus, the regulation of resection initiation and its subsequent progression are thought to determine which repair pathway, HR, NHEJ or microhomology-mediated end joining^6^, acts to repair the break.

DSB repair and its regulation occur on chromatin. A cascade of proteins associate with chromatin in proximity to a DSB and this is largely dependent on H2AX phosphorylation by DNA damage signalling proteins^7^. This accumulation of proteins into foci has been suggested to serve a number of specific functions; increasing the local concentration of repair factors, allowing amplification of DNA damage signals and facilitating the regulation of resection to promote the use of the appropriate DNA repair pathway^2^. In addition to H2AX phosphorylation, multiple other histone modifications have been directly linked to DSB signalling and repair in both yeast and human cells^8^, but many details regarding how chromatin modification coordinates with the signalling, processing and repair of DNA damage remain elusive.

The Cullin 4 ring E3 ubiquitin ligases (CRL4s) regulate a wide variety of biological processes including chromatin remodelling, DNA replication and repair^9^. Like SCF (Skp1-CUL1-F box) complexes, CRL4s consist of a core set of components (CUL4-DDB1-ROC) associated with one of a variety of substrate-specific adaptors that target defined proteins to CRL4 for ubiquitin-dependent modification. These adaptors form a family of ‘DWD' (DDB1-binding WD40) proteins that define multiple CRL4 complexes (that is, CRL4^Cdt2^; CRL4^Ddb2^; and so on)^9^. Database search and biochemical analysis predict that about one-third of the human WD40 proteins contain DWD motif and thus are prospective CRL4 targeting subunits. Only few of these have been biologically characterized.

Fission yeast CRL4^Cdt2^ was shown to regulate dNTP synthesis during DNA replication and repair by promoting degradation of Spd1, a ribonucleotide reductase (RNR) inhibitor^10^,^11^,^12^,^13^. Deletion of *spd1* can partially alleviate the hypersensitivity of *cul4*Δ and *ddb1*Δ mutants to genotoxic agents, but does not fully reverse these phenotypes^11^. This indicated that CRL4s regulate the DNA damage response via additional substrate-targeting subunits. Based on this hypothesis, we set out to identify additional roles for CRL4s in the DNA damage response. We identified Wdr70, a previously uncharacterized WD40-repeat protein. We demonstrate that Wdr70 associates with Ddb1 to form a novel CRL4 complex and that the level of this complex is increased in response to DNA damage. We establish that CRL4^Wdr70^ specifically promotes resection of DSBs and HR by regulating the monoubiquitination status of histone H2B (uH2B). We show that this mechanism is evolutionarily conserved in human cells, identifying a novel aspect of chromatin regulation during DSB repair.

## Results

### Wdr70 forms a damage-induced CRL4 complex

**Figure 1:**
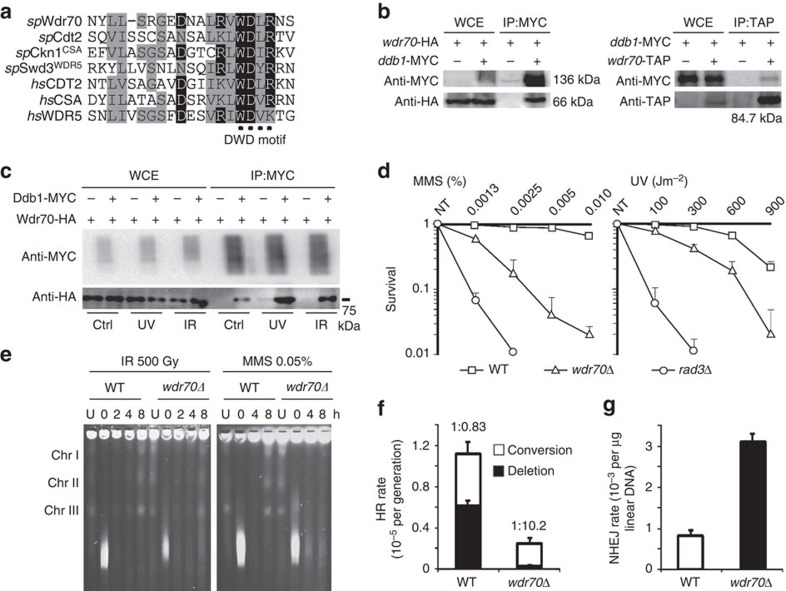
CRL4^Wdr70^ influences DSB repair pathway selection. (**a**) The putative DWD motif of Wdr70. (**b**) Co-immunoprecipitation (co-IP) of tagged Ddb1 and Wdr70. MYC, TAP and HA-tagged proteins were expressed from their genomic loci. (**c**) Co-IP as in **b** following ultraviolet (UV; 500 J m^−2^) or ionising radiation (IR; 80 Gy) treatment. (**d**) Clonogenic survival analysis of *wdr70*^+^ and *wdr70*Δ cells following MMS or UV exposure. *rad3*Δ was used as a hypersensitive control. *n*=3 biological repeats. Error bars=s.d. (**e**) Pulse field electrophoresis of genomic DNA extracted from *wdr70*^+^ and *wdr70*Δ cells at the indicated time points after MMS or IR treatment. (**f**) Comparative analysis of HR efficiency between *wdr70*^+^ and *wdr70*Δ cells (*spd1*Δ background) using a heteroallelic recombination assay. Ratios represents the proportion of conversion (*ade*^+^ *his*^+^) to deletion (*ade*^+^ *his*^−^) type repair products. *n*=3 biological repeats. HR, homologous recombination. Error bars=s.d. (**g**) Comparative analysis of NHEJ efficiency using a plasmid re-joining assay (*spd1*Δ background). *n*=3 biological repeats. NHEJ, non homologous end joining. Error bars=s.d.

Fission yeast CRL4^Cdt2^ regulates dNTP synthesis during DNA replication and repair by promoting degradation of Spd1, a RNR inhibitor^11^,^12^,^13^. To identify additional roles for CRL4s in the DNA damage response, we introduced a cDNA library into *ddb1*Δ *spd1*Δ cells and screened for altered genotoxin sensitivity. We identified *wdr70*^+^ (SPAC343.17c), expression of which increased sensitivity to a range of treatments (see Supplementary Fig. 1a for details). Wdr70 is a conserved nuclear WD40-repeat protein with a ‘DWD' motif (^325^WDxR^328^; Fig. 1a and Supplementary Fig. 1b,c) that defines the association between DCAF and the CRL4 subunit, Ddb1 (ref. 14). We showed that Wdr70 physically interacts with Ddb1 (Fig. 1b) in a DWD-dependent manner (Supplementary Fig. 1d), that this interaction is resistant to ethidium bromide (Supplementary Fig. 1e) and enhanced upon ultraviolet light treatment or exposure to ionising radiation (IR) (Fig. 1c). This indicates the formation of a CRL4^Wdr70^ complex that is DNA damage stimulated.

*wdr70* null cells (*wdr70*Δ), or cells harbouring a mutation in the DWD motif that prevents binding to Ddb1 (*wdr70-wd*), exhibited slow growth and an elongated cell phenotype typical of delayed G2/M progression (Supplementary Fig. 1f,g). These phenotypes are reminiscent *S. pombe* DNA repair mutants that accumulate unrepaired spontaneous DNA damage^15^. Consistent with increased endogenous lesions, *wdr70*Δ viability was dependent on *rad3*^ATR^, a core DNA structure checkpoint gene, and untreated *wdr70*Δ cells showed low plating efficiency, a high incidence of mitotic catastrophe and elevated spontaneous Rad52 foci (Supplementary Fig. 2a–d). When compared with *wdr70*^+^ controls, *wdr70*Δ cells displayed viability loss upon ultraviolet or Methyl methanesulfonate (MMS) treatment (Fig. 1d). Although not sensitive to IR and CPT treatment (Supplementary Fig. 2e), *wdr70*Δ cells showed prolonged Chk1 phosphorylation and enhanced mitotic delay in response to treatment with both IR and MMS (Supplementary Fig. 2f). Repair capacity, as judged by recovery of full-length chromosomes upon pulse field gel electrophoresis, was reduced in response to MMS treatment, but not IR treatment (Fig. 1e).

CRL4^Cdt2^ degrades Spd1 to regulate RNR to promote nucleotide synthesis for postsynaptic gap filling and efficient HR^13^. We observed that Spd1 levels are increased in *wdr70*Δ G2 cells (*spd1* mRNA levels are elevated) and that, consistent with this, concomitant *spd1* deletion partially rescued *wdr70*Δ damage sensitivity (Supplementary Fig. 2g). To ensure the Wdr70-dependent DNA damage response phenotypes that we characterize below were independent of Spd1-mediated dNTP regulation, we performed all further genetic analysis in *spd1*Δ backgrounds. Unless otherwise stated as *spd1*^+^, all strains labelled as ‘WT' are *spd1*Δ.

### Wdr70 promotes long-range resection at DSB

**Figure 2:**
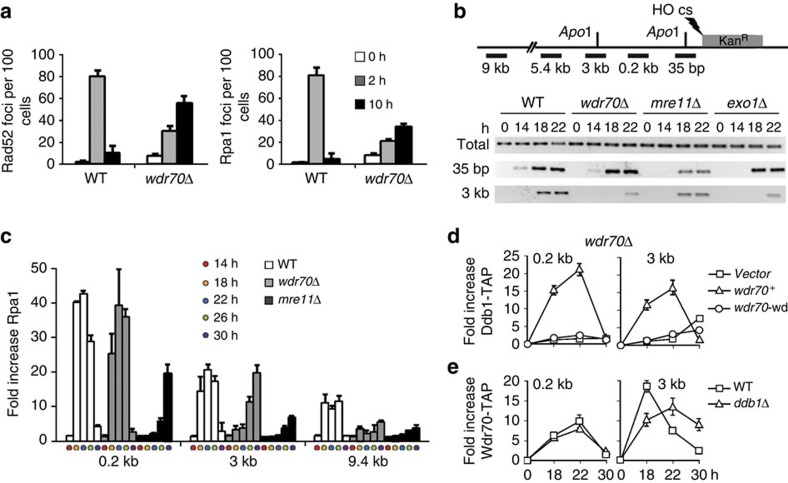
*wdr70* mutants are defective in DSB resection. All genetic backgrounds are *spd1*Δ. (**a**) Formation of Rad52 and Rpa1 foci after 80 Gy ionizing radiation in *wdr70*^+^ cells and *wdr70*Δ cells. *n*=3 biological repeats. Error bars=s.d. (**b**) Top: schematic representation of the HO-DSB locus: vertical lines: *Apo*I sites (35 bp and 3 kb from break site). HO cs: HO cut site; Kan^R^; kanamycin resistance gene; black bars, PCR amplicons. Bottom: resection was assessed by PCR across the *Apo*I sites following digestion in the indicated strains. Samples were taken at time points shown following thiamine removal. HO induction from the *nmt41* promoter requires ∼14 h to initiate. Loading was normalized by amplification of an uncut region (total). (**c**) Rpa1 recruitment was assayed by chromatin immunoprecipitation (ChIP) at 0.2, 3 and 9.4 kb from the DSB in *wdr70*^+^, *wdr70*Δ and *mre11*Δ backgrounds. Vertical axis: fold enrichment relative to *T*=0. *n*=3 biological repeats. Error bars=s.d. (**d**) Ddb1 recruitment measured by ChIP in *wdr70*Δ cells transformed with empty Rep41 vector (V), *wdr70*^+^ or *wdr70*-W325A/D326A (*wdr70*-*wd*) plasmids. (**e**) Recruitment of Wdr70 was measured by ChIP in the indicated strains 0.2 and 3 kb from the DSB site.

The mechanism of spontaneous DNA break repair can be assessed in *S. pombe* using a heteroallelic recombination assay in which non-tandem direct repeats of *ade6* heteroalleles can revert to ade^+^ by either gene conversion or a recombination event that deletes the intervening sequence^16^. When compared with *wdr70*^+^ controls, spontaneous HR-dependent gene conversion and deletion was suppressed in a *wdr70*Δ background (Fig. 1f and Supplementary Fig. 2h). Consistent with this, in a plasmid re-joining assay outcomes associated with NHEJ were elevated 3.7-fold (Fig. 1g). This indicated that Wdr70 functions to promote HR repair of DSBs and, in its absence, NHEJ becomes more central. We next characterized the kinetics of HR by monitoring IR-induced Rpa1 and Rad52 foci. Peak levels of Rpa1 and Rad52 foci were delayed in *wdr70*Δ when compared with *wdr70*^+^ cells: extensive foci remained in *wdr70*Δ cells 10 h after treatment, whereas foci in *wdr70*^+^ cells were resolved by 4 h (Fig. 2a and Supplementary Fig. 3a,b). This suggested that resection was delayed in the *wdr70*Δ background. To explore this further, we induced a single DSB using the HO endonuclease and monitored resection by PCR. Resection results in ssDNA that is refractory to restriction endonuclease digestion. Thus, following digestion, resected DNA remains intact for amplification. Two *Apo*I sites (Fig. 2b) were assayed, one immediately proximal (35 bp) to the HO-induced DSB site, the other distal (3 kb) from the break^17^.

In *wdr70*^+^ cells, DSB resection was apparent both immediately proximal and distal to the break. In contrast, in either *wdr70*Δ or *exo1*Δ control cells, resection 3 kb from the break was impaired (Fig. 2b). In this semi-quantitative assay, *mre11*Δ cells displayed a reduction in both proximal and distal resection, as expected. To confirm that Wdr70 affected long-range resection, we performed Rpa1 chromatin immune-precipitation (ChIP). Rpa1 associated with DNA proximal (0.2 kb) to the DSB with similar kinetics for both *wdr70*^+^ and *wdr70*Δ cells but delayed association was evident distal (3 and 9.4 kb) to the break in *wdr70*Δ (Fig. 2c). *mre11*Δ cells were, as expected, partially defective in both proximal and distal Rpa1 association.

### Wdr70 mediates uH2B spreading by RNF20-RNF40 (HULC^Rhp6^)

To determine if the observed defect in resection was due to a direct role for Wdr70 or was an indirect consequence of other changes to cell metabolism, we used ChIP analysis to establish if CRL4^Wdr70^ subunits were recruited to sites of DNA breakage. Both Wdr70 and Ddb1 were found to directly associate both proximal (0.2 kb) and distal (3 kb) to an induced DSB site (Fig. 2d,e and Supplementary Fig. 3c,d). Loss of Ddb1 association in the *wdr70*Δ background could not be complemented by w*dr70-wd* mutant expression (Fig. 2d), indicating that Wdr70 recruits CRL4^Wdr70^. As the COP9-Signalosome is a known regulator of CRL4 ubiquitin ligase activity^18^, we tested the requirement for the Signalosome subunits Csn1 and Csn5 in Wdr70-mediated resection. Both the *csn1*Δ and *csn5*Δ mutations reduced the amount of ssDNA present distal to the induced DSB, indicating a resection defect (Supplementary Fig. 3e). These phenotypes were not additive with *wdr70*Δ when the mutations were combined in the same cells, consistent with the Wdr70-dependent regulation of resection being mediated by ubiquitin ligation.

**Figure 3:**
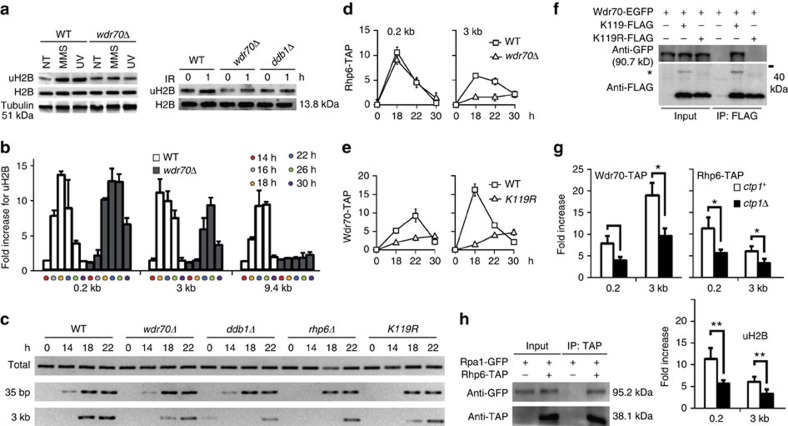
CRL4^Wdr70^ stimulates spreading of uH2B at a DSB. All genetic backgrounds are *spd1*Δ. (**a**) Western blot of uH2B levels in the indicated strains following DNA damage (NT; no treatment). (**b**) uH2B enrichment at the 0.2, 3 and 9.4 kb from a DSB assayed by chromatin immunoprecipitation (ChIP) in *wdr70*^+^ and *wdr70*Δ backgrounds. *n*=3 biological repeats. Error bars=s.d. (**c**) Resection assessed at the indicated times by PCR across the *Apo*I sites (see Fig. 2b) following *Apo*I digestion in *wdr70*^+^ (*spd1*Δ), *wdr70*Δ, *ddb1*Δ, *rhp6*Δ and H2B-*K119R* backgrounds. (**d**) Recruitment of Rhp6 in *wdr70*^+^ (WT) and *wdr70*Δ cells 0.2 and 3 kb from the break site assayed by ChIP. *n*=3 biological repeats. Error bars=s.d. (**e**) Recruitment Wdr70 assayed by ChIP at 0.2 and 3 kb from the break site for wild-type and *H2B-K119R* mutant cells. *n*=3 biological repeats. Error bars=s.d. (**f**) co-IP of EGFP-tagged Wdr70 and Flag-tagged H2B (K119 WT or K119R). Cell lysates were obtained from MMS-challenged cells, treated with ethidium bromide for 10 min and precipitated by anti-Flag antibody. * Indicates Ub-H2B. (**g**) Chromatin association of Wdr70, Rhp6 and uH2B at 0.2 and 3 kb from the break site assayed by ChIP for *ctp1*^+^ and *ctp1*Δ. *n*=2 biological repeats each with three technical repeats. *P<0.05; **P<0.01 (*t*-test). (**h**) co-IP of Rpa1-GFP with Rhp6-TAP. Method as described in **f**.

Because CRL4s have previously been linked to the regulation of histone dynamics^19^,^20^,^21^ and chromatin status is known to influence DSB processing^22^, we systematically screened known histone modifications in IR-treated *wdr70*^+^ and *wdr70*Δ cell lysates (Supplementary Fig. 4a,b). The major variation we observed was H2B lysine 119 mono-ubiquitination and we found that induction of uH2B by DNA damage was partially dependent on *wdr70* or *ddb1* (Fig. 3a). H2B monoubiquitination is catalysed by the RNF20–RNF40–UbcH6 complex^23^ (known as HULC^Rhp6^ in *S. pombe*^24^) and promotes chromatin remodelling during transcription by influencing H3 methylation^25^. RNF20-dependent H2B monoubiquitination has also been linked to the promotion of DSB repair through the regulation of resection and the recruitment of HR factors in human cells^26^,^27^.

Consistent with H2B monoubiquitination functioning at DNA repair sites, we found that uH2B in *S. pombe* was enriched both proximal (0.2 kb) and distal (3 and 9.4 kb) to an induced DSB with kinetics similar to Rpa1 (Fig. 3b). To explore the role of CRL4^Wdr70^ in the regulation of uH2B at DNA damage sites, we examined uH2B induction in the *wdr70* mutant backgrounds. Either deletion of *wdr70* (Fig. 3b) or a mutation of the DWD motif (Supplementary Fig. 4c) reduced uH2B enrichment at sequences distal to an induced DSB but not at the proximal sequences. As *rhp6* is required for uH2B enrichment (Supplementary Fig. 4d), we propose that CRL4^Wdr70^ is not a ligase for H2B-K119 ubiquitination, but regulates H2B ubiquitination by HULC^Rhp6^.

If the resection defect observed in *wdr70*Δ cells is caused by failure to regulate uH2B, then single-stranded DNA production around an induced DSB should be similarly affected in *rhp6*Δ cells (the E2 for HULC^Rhp6^)^24^ as well as in cells harbouring H2B-K119R mutations. Both *rhp6*Δ and *H2B-K119R* cells displayed attenuated ssDNA at DSB-distal sites (Fig. 3c). We also observed that the double *H2B*-*K119R wdr70*Δ mutant cells demonstrated epistasis for genotoxic treatments (Supplementary Fig. 4e), consistent with CRL4^Wdr70^ regulating H2B monoubiquitination by HULC^Rhp6^. Further support for CRL4^Wdr70^ regulating uH2B, but not directly modifying H2B itself, comes from the observation that concomitant deletion of *wdr70* and *ubp8* (an uH2B-specific de-ubiquitinase^28^) partially rescued the *wdr70*Δ-dependent defects in uH2B and MMS survival (Supplementary Fig. 4f,g), whereas *ubp8* deletion did not rescue *rhp6*Δ sensitivity (Supplementary Fig. 4g). Using RNA sequencing profiling of genome-wide gene expression (Supplementary Table 1), we did not observe changes to the expression of genes implicated in resection. Similarly, assessing the protein levels of a variety of repair factors (Supplementary Fig. 4h) did not identify significant changes. Taken together, our data thus suggest that CRL4^Wdr70^ functions to regulate Rhp6-dependent H2B ubiquitination surrounding DSBs in order to facilitate long-range resection.

To further explore the role of Wdr70 in establishing uH2B chromatin domains at DSB sites, we monitored the recruitment of the HULC^Rhp6^ complex subunits. Rhp6 E2 ligase recruitment was evident both proximal (0.2 kb) and distal (3 kb) in *wdr70*^+^ cells, whereas distal but not proximal recruitment was reduced in *wdr70*Δ cells (Fig. 3d). A decrease in recruitment of Brl1, the HULC^Rhp6^ complex E3, was also observed at the distal, but not the proximal location (Supplementary Fig. 4i). Interestingly, the chromatin loading of Wdr70 was decreased both break-proximal and distal in the *H2B*-*K119R* background (Fig. 3e), indicating that initial H2B monoubiquitination is required to recruit CRL4^Wdr70^ to facilitate the subsequent modification cascade. Consistent with this interpretation, Wdr70 and H2B could be co-immunoprecipitated from wild type cell extracts in the presence of ethidium bromide, but not the *H2B-K119R* cell extracts (Fig. 3f).

**Figure 4:**
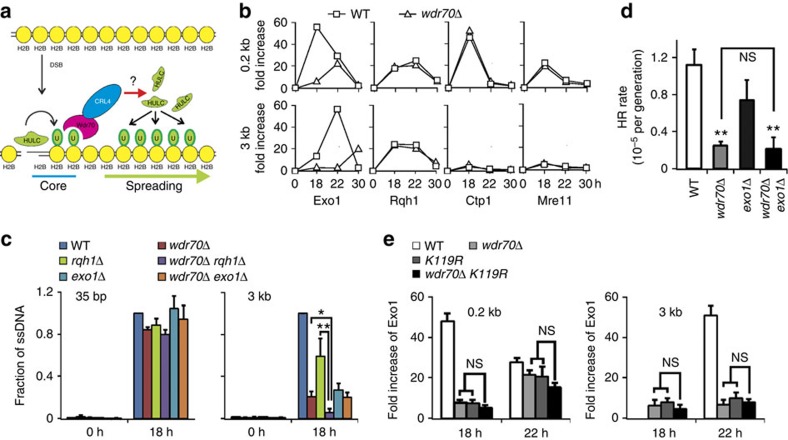
CRL4^Wdr70^ promotes recruitment of Exo1 to DSB. All genetic backgrounds are *spd1*Δ. (**a**) A two-step model for H2B mono-ubiquitination after DSB induction. CRL4^Wdr70^ facilitates the loading of HULC^Rhp6^ and the spreading of uH2B distal from the break site after formation of the HULC^Rhp6^-catalysed uH2B core proximal to the DSB. (**b**) An experiment showing the recruitment of Exo1, Rqh1, Ctp1 and Mre11 when assayed by chromatin immunoprecipitation (ChIP) in *wdr70*^+^ and *wdr70*Δ backgrounds 0.2 and 3 kb from the DSB at the indicated times after thiamine removal. HO induction from the *nmt41* promoter requires ∼14 h to initiate. WT, wild type; *n*=3. Error bars not shown. (**c**) Resection was subsequently assessed by quantitative PCR across the *Apo*I sites (see Fig. 2b) following *Apo*I digestion of DNA isolated at 0 and 18 h in the indicated strains. *n*=3 biological repeats. Error bars=s.d. *P<0.05; **P<0.01 (*t*-test). (**d**) Heteroallelic recombination assay for *wdr70*Δ, *exo1*Δ and the double *wdr70*Δ *exo1*Δ background. *n*=3 biological repeats. Error bars=s.d. **P<0.01 (*t*-test). NS, no statistical significance between strains. (**e**) Exo1 enrichment measured by ChIP 0.2 and 3 kb from an induced DSB in *wdr70*Δ, *H2B-K119R* and *wdr70*Δ *H2B-K119R* backgrounds to assess epistasis.

Resection is initiated by MRN- and Ctp1-dependent nuclease activity to generate a short ssDNA substrate. This is followed by further resection dependent on Exo1 and Rqh1^BLM^-Dna2. Our data suggest the initial short tract of ssDNA triggers proximal uH2B, with further uH2B spreading dependent on CRL4^Wdr70^. In support of this recruitment of both Wdr70 and Rhp6 plus the uH2B modification were significantly decreased both proximal and distal in *ctp1*Δ cells (Fig. 3g). Since neither CRL4^Wdr70^ nor uH2B influence proximal resection (Fig. 3c), we conclude that HULC^Rhp6^ is recruited to the initial ssDNA-RPA exposed proximal to the DSB to form a core uH2B domain. Consistent with this, we were able to co-immunoprecipitate Rhp6 and Rpa1 in the presence of ethidium bromide (Fig. 3h). Thus, our data indicate that uH2B is modified in response to a DSB in two separable stages: an initial core domain is triggered by short-range resection followed by a subsequent CRL4^Wdr70^-dependent spreading distal to the DSB (Fig. 4a).

### DSB recruitment of Exo1 nuclease by Wdr70 and uH2B

To distinguish which resection pathway Wdr70 influences, we monitored recruitment of Mre11, Ctp1, Exo1 and Rqh1^BLM^ both proximal and distal to an induced DSB (Fig. 4b). As expected, Mre11 and Ctp1 were recruited to proximal, but not distal, sites. Their profiles were not affected by loss of CRL4^Wdr70^. Rqh1^BLM^, which participates with Dna2 in long-range resection, was recruited at both proximal and distal sites and was unaffected by loss of CRL4^Wdr70^. This implies that Rqh1-dependent resection is independent of uH2B. In contrast, Exo1 recruitment at both the proximal and the distal loci was severely impaired by CRL4^Wdr70^ loss. This implies that Wdr70, and thus uH2B, mainly influences Exo1-dependent resection. Consistent with this, we observed that *rqh1*Δ *wdr70*Δ double mutant cells, but not *exo1*Δ *wdr70*Δ double mutants, displayed additive resection defects (Fig. 4c). Furthermore, *rqh1*Δ *wdr70*Δ, but not *exo1*Δ *wdr70*Δ, showed synergistic MMS sensitivity (Supplementary Fig. 5a) and concomitant deletion of *exo1* did not exacerbate the *wdr70*Δ HR deficiency as assayed by spontaneous heteroallelic recombination (Fig. 4d)

*In vitro*, resection by Exo1, as opposed to resection by the Rqh1^BLM^-Dna2 complex, is blocked by nucleosomes and removal of H2A-H2B dimers partially overcomes this^29^. We therefore tested if the decreased recruitment of Exo1 observed in *wdr70Δ* cells was epistatic to the loss of uH2B. In cells where the *wdr70*Δ and *H2B-K119R* mutants were combined, no statistically significant changes were seen for Exo1 recruitment when compared with either the *wdr70*Δ or *H2B-K119R* backgrounds (Fig. 4e). Overexpression of Exo1 did not rescue the *wdr70*Δ resection defect indicating that uH2B promotes Exo1 resection activity, rather than its initial recruitment (Supplementary Fig 5b). We next quantified the recruitment of Wdr70 and H2B monoubiquitination in *exo1*Δ, *rqh1*Δ and *exo1*Δ *rqh1*Δ double mutant cells, the latter of which are predominantly defective in long-range resection (Supplementary Fig 5c). In contrast to control and single mutant cells, the *exo1*Δ *rqh1*Δ double mutant exhibited compromised Wdr70 recruitment and H2B modification distal to a DSB. This is distinct from the observation that DSB proximal uH2B is independent of long-range resection (but is compromised in *ctp1*Δ cells, where the initial resection is defective). We conclude that Wdr70-dependent uH2B spreading from a DSB is co-operative with ongoing resection to allow access of the Exo1-dependent resection machinery.

### Wdr70 counteracts the Crb2^53BP1^-dependent resection barrier

Resection is regulated by multi-layered mechanisms, of which 53BP1 and its homologues (Crb2^53BP1^ in *S. pombe* and Rad9^53BP1^ in *S. cerevisiae*) are the most studied. In *S. pombe*, Crb2^53BP1^ associates with chromatin through co-operative binding to γH2A and di-methylated H4K20 (H4K20me2) via its BRCT and Tudor domains, respectively^30^,^31^,^32^. All H4K20me2 is dependent on the Set9 methyltransferase in fission yeast^33^.

**Figure 5:**
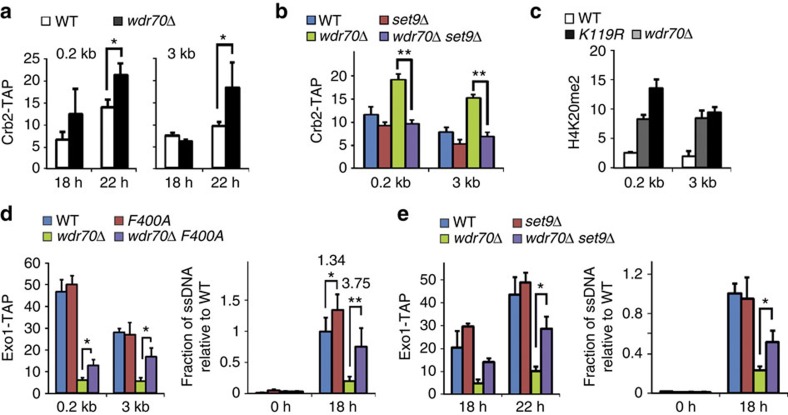
CRL4^Wdr70^ suppresses Crb2^53BP1^ loading at DSBs. All genetic backgrounds are *spd1*Δ. (**a**) Recruitment of Crb2^53BP1^ assayed by ChIP at 0.2 and 3 kb from the DSB following induction in *wdr70*^+^ (WT) and *wdr70*Δ backgrounds. (**b**) Recruitment of Crb2^53BP1^ assayed by ChIP at 0.2 and 3 kb from the DSB in *set9*Δ, *wdr70*Δ and *set9*Δ *wdr70*Δ double mutant backgrounds. (**c**) ChIP assay of H4K20me2 enrichment 0.2 and 3 kb from the DSB in *wdr70*^+^, *wdr70*Δ and *H2B-K119R* backgrounds. (**d**) Recruitment of Exo1 assayed by ChIP (left) and resection analysed by quantitative PCR (right) 3 kb distal to an HO break in the *crb2-F400A*, *wdr70*Δ and *crb2-F400A wdr70*Δ double mutant backgrounds. (**e**) An equivalent experiment as **d** for *set9*Δ, *wdr70*Δ and *set9*Δ w*dr70*Δ double mutant backgrounds. All panels: *n*=3 biological repeats. Error bars=s.d. *P<0.05, **P<0.01 (*t*-test).

In the *wdr70*Δ mutant, we observed that both proximal and distal DSB recruitment of Crb2^53BP1^ was increased when compared with *wdr70*^+^ controls (Fig. 5a). This increase was lost upon concomitant deletion of the Set9 (Fig. 5b). Consistent with Crb2^53BP1^ being recruited via H4K20me2, the level of H4K20me2 was elevated in both *wdr70*Δ and *K119R* mutants both proximal and distal to the DSB (Fig. 5c and Supplementary Fig. 5d). This did not correlate with a global increase in H4K20me2 (Supplementary Fig. 5e) consistent with previous studies showing that H4K20me2 is not synthesized *de novo* after DNA damage^33^,^34^. It has previously been reported that a missense mutation in the Crb2^53BP1^ Tudor domain, F400A, ablates Crb2^53BP1^ binding to H4K20me2 (ref. 32). The presence of the *crb2-F400A* mutation partially rescued the *wdr70*Δ-dependent recruitment defect of Exo1 both proximal and distal to the induced DSB site (Fig. 5d). The levels of ssDNA 3 kb distal to the DSB was similarly rescued (Fig. 5d). An equivalent rescue of the Exo1 recruitment defects seen in *wdr70*Δ mutants was also evident when s*et9* was deleted (Fig. 5e). Again, this correlated with rescue of the *wdr70*Δ-dependent resection defects (Fig. 5e).

Mre11-dependent nuclease activity is required to dissociate Ku70-Ku80 from DSB ends and in the *mre11*Δ mutant background the inability to release Ku interferes with resection and RPA recruitment^17^. Concomitant Ku deletion thus restores proximal RPA recruitment in the *mre11*Δ mutant. Unlike the effects of *crb2*Δ and *set9*Δ, concomitant *pku70* deletion in *wdr70*Δ cells did not restore ssDNA formation 3 kb from the DSB (Supplementary Fig. 5f). This indicates that Wdr70 does not participate in removing Ku from DSB ends. Taken together, these results suggest that CRL4^Wdr70^-uH2B counteracts a Crb2^53BP1^-dependent barrier to Exo1 recruitment and resection.

### Wdr70 promotes resection in human cells

**Figure 6:**
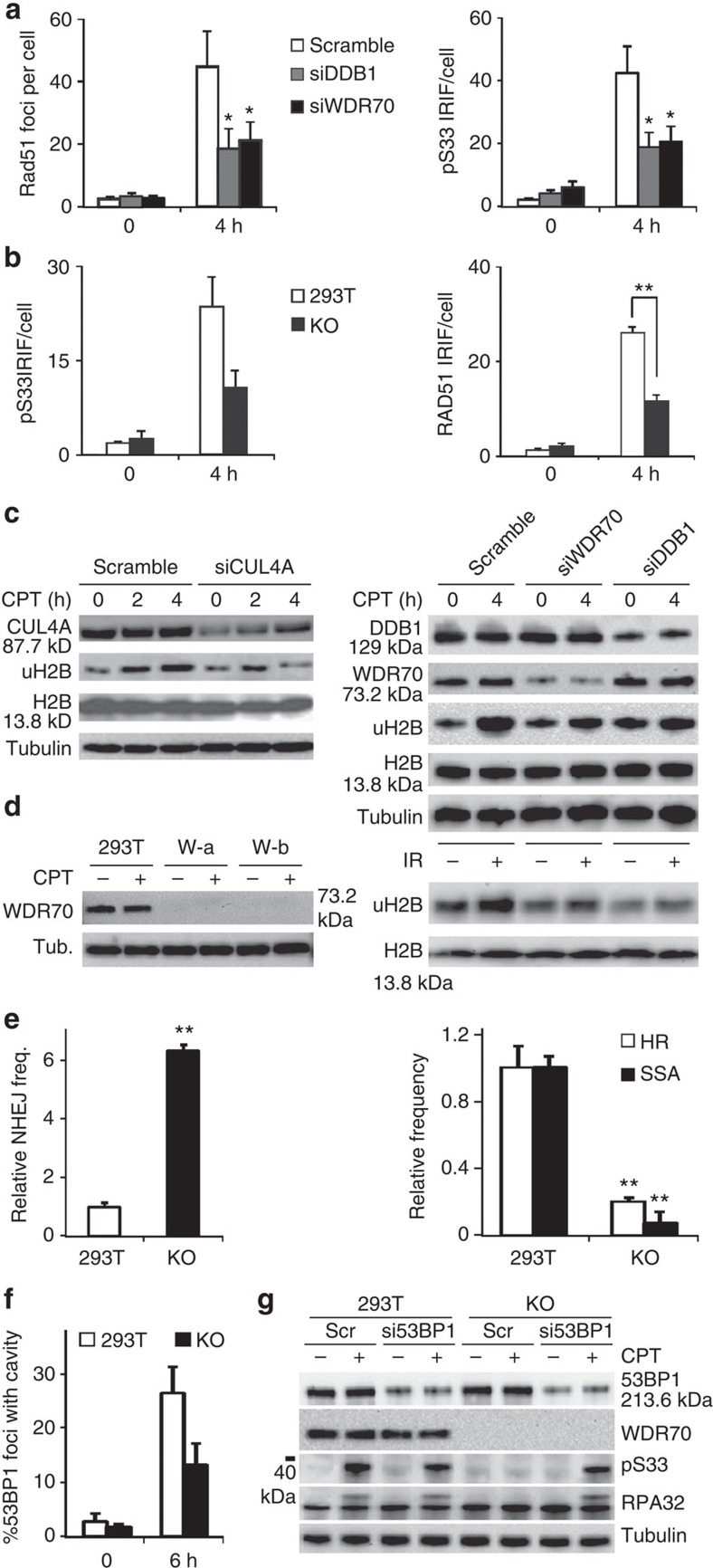
Prolonged occupancy of 53BP1 upon ablation of mammalian CRL4^WDR70^. (**a**) Quantification of Rad51 and phospho-Rpa32 (pS33) foci in 293T cells transfected with DDB1 or WDR70 siRNA. *n*=3 biological repeats. Error bars=s.d. *P<0.05, *t*-test. (**b**) CRISPR knockout (KO) WDR70 cells are defective for Rad51 IRIF and ionising radiation (IR)-induced RPA32-S33 phospho foci. *n*=3 biological repeats. Error bars=s.d. **P<0.01, *t*-test. (**c**) Immuno-detection of damage-induced H2B ubiquitination in 293T cells either pre-treated with siRNA specific for human CRL4^WDR70^ genes or a scrambled control. (**d)** Western blot of two independent WDR70 KO cell lines generated by CRISPR. W-b was used for subsequent experiments. (**e**) Quantification of repair efficiency for I-*Sce*I-induced DSB using quantitative PCR following transfection of linearized plasmids into parental and WDR70 KO 293T cells. *n*=3 biological repeats. Error bars=s.d. **P<0.01, *t*-test. (**f**) Quantification of 53BP1 foci with obvious cavities 6 h following IR treatment. *n*=3 biological repeats. Error bars=s.d. (**g**) Immunoblot for CPT-induced Rpa32 phosphorylation in 293T and WDR70 KO cells treated with either control or 53BP1 siRNA.

As with yeasts, metazoan 53BP1 is recruited to DSBs where it inhibits resection and promotes NHEJ^35^,^36^,^37^. We assessed the conservation of the CRL4^WDR70^ DNA repair function in human cells by depletion of either DDB1 or WDR70 using short interfering RNA (siRNA) on HEK293T cells. Both DDB1 and WDR70 depletion impaired resection, as judged by the attenuation of ionizing irradiation-induced foci for Rad51, RPA32 and RPA32-S33 phosphorylation (Fig. 6a,b and Supplementary Fig 6a–d). Cells deficient for either DDB1 or WDR70 were also sensitive to treatment with ultraviolet, CPT or IR (Supplementary Fig. 6e). We next examined the induced levels of uH2B. The increase of total uH2B that is observed in response to treatment with genotoxic agents was attenuated upon CPT treatment of cells in which CUL4A, DDB1 or WDR70 were depleted by siRNA treatment when compared with a control cells treated with scrambled siRNA. A similar trend was evident after IR treatment for cells depleted of DDB1 or WDR70 (Fig. 6c). This suggests that, in human cells as in *S. pombe*, CRL4^Wdr70^ participates DNA damage-dependent uH2B and the regulation of resection.

Using an I-*Sce*I-mediated DSB system^38^ we found that, when WDR70 was inactivated by CRISPR technology (Fig. 6d), NHEJ usage increased and HR and SSA were compromised (Fig. 6e). In mammalian cells, 53BP1 is usually expelled from the core of repair centres 4–8 h post-IR, forming a ‘cavity' that is filled by subsequent RPA recruitment^39^. We thus examined, 6 h post irradiation, the morphology of 53BP1 foci and their co-localization with phospo-RPA32 foci. The appearance of cavities at the foci centres (Fig. 6f) and the recruitment of phospho-RPA32 was delayed in WDR70 knockout cells when compared with the control (Supplementary Fig. 6f). Consistent with this, depletion of 53BP1 by siRNA partially rescued the increase in NHEJ observed in WDR70 knockout cells (Supplementary Fig. 6g). Finally, in cell extracts, the RPA32 phosphorylation observed in response to camptothecin treatment was attenuated in WDR70 knockout cells. This attenuation was reversed by the transfection of 53BP1 siRNA (Fig. 6g). Consistent with reports that H4K20Me2 is not induced in response to DNA damage^34^, we did not observe changes to H4K20Me2 levels in the absence Wdr70 (Supplementary Fig. 6h). Collectively, these data are reminiscent of our observations in *S. pombe*: impaired DSB repair correlates with impaired Crb2^53BP1^ repositioning and defects in RPA association with damaged DNA.

## Discussion

DNA damage signalling and DNA repair occur in the context of chromatin and are modulated by multiple pathways that orchestrate spatio-temporal histone modifications to promote signalling and allow access of appropriate repair factors to DNA lesions^40^. A key posttranslational modification is the C-terminal phosphorylation of a H2AX (H2A in yeast), which initiates a cascade of chromatin-associated posttranslational modifications that modulate protein localization and activity in the vicinity of the DNA damage. However, not all histone modifications are dependent on prior H2AX phosphorylation. For example, H4K20 di-methylation occurs independently^26^,^27^, suggesting parallel activities converge to correctly regulate the overall DNA damage response.

DNA DSBs are the most genotoxic form of DNA damage as a single DSB can result in gross chromosomal rearrangement or cell death. Unsurprisingly, multiple DNA repair pathways have evolved to repair DSBs. HR is largely error free, while other pathways such as NHEJ and alternative micro-homology mediated end-joining pathways are intrinsically error prone. The choice of pathway used is thus at least partly dictated by the availability of an appropriate homologous template: in S phase and G2, the sister chromatid is close by due to cohesion, whereas in G1 the homologous chromosome is likely far away and thus the risk of translocation increased. Multiple mechanisms regulate the choice of repair pathway, responding to cues including the activity of cyclin-dependent kinases^3^,^41^ and the chromatin environment in which the damage arose^42^. Various histone modifications impact on this pathway choice, for example, in human cells H2AX-dependent RNF8-RNF168 ubiquitination of H2A promotes 53BP1 association, which inhibits DSB processing and favours NHEJ unless this inhibitory activity is countered by BRCA1 (ref. 2). The parallel pathway of H2B ubiquitination by RNF20-RNF40-UbcH6 (HULC^Rhp6^ in fission yeast) is necessary for DNA resection and efficient HR^26^,^27^. Precisely, how each of the plethora of pathways and proteins that affect DNA processing at a DSB interact is the subject of much ongoing research.

In exploring how CRL4s might promote efficient recovery from DNA damage, we uncovered evidence that CRL4^Wdr70^ acts as a chromatin modulator that modulates the extent of H2B monoubiquitination. In turn, this affects the extent to which DSBs are resected. We show that although uH2B occurs immediately proximal to the break site in the absence of CRL4^Wdr70^, the complex is necessary for distal uH2B several kilobases from the DSB site. Thus, we propose that CRL4^Wdr70^ does not itself modify H2B but, through presumed modification of an unknown target, acts to modulate the activity of HULC^Rhp6^. A lack of distal uH2B, or the complete loss of uH2B, results in impaired Exo1-dependent resection. This correlates with a decrease in HR and an increase in the use of end joining pathways of repair. Intriguingly, these defects can be reversed by the concomitant deletion of Crb2^53BP1^, which forms a barrier for resection.

Loss of CRL4^Wdr70^ function impacts on the sensitivity of *S. pombe* to various genotoxins including MMS and ultraviolet light, indicating that the regulation of resection is important when lesions are encountered in S phase (Fig. 1d). Unexpectedly, sensitivity to IR, which results in the majority of breaks being formed in G2 cells in fission yeast, is not increased in *wdr70* mutant cells (Supplementary Fig. 2e) and no gross defects in IR-induced DSB repair are seen by pulse-field gel electrophoresis (PFGE) analysis (Fig. 1e). We speculate that this is because slow resection can be compensated for in these circumstances by an extended G2/M arrest (Supplementary Fig. 2f) that provides additional time for repair and, potentially, by the use of alternative DNA repair pathways. In human cells, depletion of Wdr70 does result in a modest sensitivity to IR (Supplementary Fig. 6e) as well as to other genotoxic agents. This suggests that the reduction of distal uH2B and the consequent attenuation of Exo1-dependent resection caused by CRL4^Wdr70^ loss cannot be fully compensated for in mammalian cells.

Exactly how uH2B facilitates access of the Exo1 resection machinery is unknown. *In vitro*, Exo1 is refractory to chromatin, unlike the BLM-Dna2 resection machinery^29^, and this correlates with our observation that Exo1, but not Rqh1^BLM^-Dna2-mediated resection, is attenuated when H2B monoubiquitination is perturbed (Fig. 4). However, we cannot explain why Crb2^53BP1^ loss should allow access to Exo1 and restore resection in the context of defective H2B monoubiquitination. It is possible that Crb2^53BP1^ binds tightly to chromatin, thus locking it in an inaccessible state that is reversed by uH2B. It is also currently unclear how uH2B facilitates chromatin changes: it is reported that nucleosomes in which H2B is monoubiquitinated, unlike those where H2A is monoubiquitinated, interfere with the compaction of 30 nm fibres^43^, potentially leading directly to a more open chromatin state. Similarly, the influence of uH2B on additional histone modifications, as is evident for H3 trans-modification during transcription^44^, may influence chromatin architecture. Alternatively, the ubiquitin moiety could act as a docking platform for a chromatin remodelling factor or factors that disrupt nucleosomes ahead of Exo1.

Our work adds a new player to the mechanisms that modulate long-range resection at the level of chromatin (Supplementary Fig. 6i). Using fission yeast, we show that both Ddb1, a core subunit of CRL4s, and Wdr70, one of many CRL4 adaptor proteins, interact in a DNA damage-inducible manner and have epistatic effects on resection suggesting that they can function in a common CRL4^Wdr70^ complex. Our analysis demonstrates that CRL4^Wdr70^ coordinates with HULC^Rhp6^ (RNF20-RNF40-UbcH6) to modulate chromatin structure at DSBs and the activity of resection enzymes, although we cannot rule out additional independent roles for Ddb1 or Wdr70 in regulating DNA repair. We further demonstrate that H2B monoubiquitination is a two-step process: HULC^Rhp6^ catalyses break-proximal uH2B on the immediate ssDNA-RPA filament formed when resection is initiated, whereas CRL4^Wdr70^ promotes subsequent HULC^Rhp6^-dependent distal H2B modification. Intriguingly, H2B monoubiquitination is coordinated with changes to H4K20me2 modification. The binding of the tudor domain of Crb2^53BP1^ to H4K20me2 is one of the mechanisms by which this inhibitor of resection is recruited to chromatin. It is postulated that H4K20 di-methylation is not synthesized *de novo* upon DNA damage, but is exposed upon changes to chromatin configuration^33^,^34^. It will be intriguing to clarify if uH2B affects the accessibility of H4K20me2.

In summary, our findings uncover a new and evolutionarily conserved histone regulatory module that influences DSB resection, HR and genome stability.

## Methods

Uncropped blots are presented in Supplementary Fig. 7. *S. pombe* strains used are listed in Supplementary Table 2. Other reagents, including antibodies and primers, are listed in Supplementary Tables 3–5.

### Yeast growth and molecular biology

Standard *S. pombe* growth media were used^45^. Gene disruption and TAP-, HA- or MYC-tagged strains were created using PCR-based HR^46^. Cells were grown in YEA or EMM-media supplemented with essential amino acids unless stated otherwise. Cell cycle synchronization was achieved by arresting *cdc25-22* cells at 37 °C for 6 h. G2 phase-arrested cells were subsequently release at 25 °C by quick cooling in ice water^47^. Septation was determined by fixing cells in methanol and staining with 4′,6-diamidino-2-phenylindole and calcofluor^45^. The w*dr70* gene was amplified from an *S. pombe* cDNA library (primers 27/28) and cloned into the *Sal*I/*Bam*HI sites of Rep41-EGFP using an infusion PCR kit (Clontech, ST0345). PFU polymerase (STRATAGENE, 600252-52) was used for site-directed mutagenesis (*W325A D326A*: primers 25/26) in Rep41-EGFP-Wdr70.

### Survival assays

To measure the sensitivity to chronic exposures to genotoxic agents, log phase cultures were harvested, resuspended in fresh media at 1 × 10^5^ per ml and diluted tenfold. 10 μl aliquots serially of these dilutions were spotted onto YEA agar plates containing indicated amounts of CPT or MMS. Representative pictures were chosen from two independent analyses. For exposure to IR, cells were irradiated using a ^137^Cs source or custom-made X-ray machine (Wandong Ltd) with indicated doses and then serially diluted and plated onto YEA plates. In the case of ultraviolet treatment, cells were serially diluted and spotted onto YEA plates and irradiated using a Stratagene Stratalinker ultraviolet source. All plates were incubated for 4–5 days at 30 °C before being photographed. Colony formation assays were carried out using the same genotoxic agents, except that 500–1,000 cells were plated onto each YEA plate and counted for colony numbers after incubation at 30 °C for 10 days. Three independent analyses were performed and each time included three plates for each concentration of drugs or radiation dosage.

### Whole-cell lysates (WCE) from *S. pombe* and western blotting

For WCE, overnight logarithmically growing yeast cell cultures in rich medium were harvested either before or after the indicated treatment with genotoxic agents or after HO-break induction. Twenty OD_600_ of cells were pelleted and resuspended in 200 μl 0.35 M NaOH for 10 min at room temperature. Two volumes of 20% trichloroacetic acid solution was added to lysed cells to precipitate protein followed by centrifugation at 20,000*g* for 10 min. Pellets were resuspended in SDS sample loading buffer (pH8.0) and boiled for 5 min. Samples were centrifuged for 1 min at 20,000*g* and supernatant was retained as WCE^48^. Boiled WCEs, histone extract or immunoprecipitated samples were resolved by 8–15% SDS–PAGE and transferred onto PVDF membrane (Roche, 03010040001) pre-soaked in methanol. Primary antibodies (listed in Supplementary Table 3) were diluted to the recommended concentration according to the manufacturer's instruction. HRP-conjugated anti-mouse or anti-rabbit IgG were obtained from DAKO. Blots were developed using Chemi docXRS (Bio-Rad). Western blots were repeated two or three times from independent experiments.

### Co-immunoprecipitation

To access the potential interactions between Wdr70 and Ddb1 or uH2B, co-IP was conducted. Briefly, 200 OD_600_ of yeast cells were lysed using a Ribolyser in IP2 lysis buffer (100 mM NaCl, 10 mM Tris-HCl, pH7.5, 5 mM EDTA, 0.5% NP-40 plus protease Cocktail (Roche, 11836170001) and ubiquitin hydrolase inhibitors (NSC632839 hydrochloride, Tocris Bioscience, 2647). Supernatants were clarified by centrifugation at 20,000*g* and incubated with 2–4 μg of anti-Myc or anti-Flag antibody for 2 h at 4 °C, followed by overnight incubation with 10 μl of Protein G DynaBeads (Invitrogen, 10004D). Captured protein complexes on the bead were subjected to extensive washing (minimum five washes) and immunoprecipitated proteins were eluted by boiling beads in SDS sample buffer for 5 min. To exclude indirect co-precipitation due to concomitant chromatin association, 10 μg of ethidium bromide was added to pre-cleared cell lysates and incubated on ice for 10 min before immunoprecipitation using 5 μg of anti-Myc antibody.

### Microscopy

To assay foci of Rad52-YFP and Rpa1-YFP, 10 OD of logarithmically growing cells collected at indicated time points after X-ray treatment were fixed in 70% ethanol for 10 min, washed in PBS and then treated with 0.5 mg ml^−1^ Zymolyase 20 T (ZYMO Research, E1004-A) in PBS for 10 min. Cells were blocked in PBS containing 10% fetal calf serum for 1 h and incubated overnight with a 1:100 dilution of anti-green fluorescent protein antibody (Roche, 1814460001) in blocking buffer containing 0.05% Tween-20. Cells were then washed in PBS and incubated with anti-mouse-FITC at 1:200 in blocking buffer containing 0.05% Tween-20 for 2 h. After washing and resuspension in PBS, cells were applied to coverslips for microscopy. DNA was visualized with 4′,6-diamidino-2-phenylindole. Three independent assays were carried out and 100 cells from more than three fields were counted for each experiment. Foci were observed and counted using an Olympus BX51 microscope and Image-Pro plus 6.0 software.

### Measurement of efficacies of NHEJ

The efficiency of NHEJ was assessed by *in vivo* circulation of Rep41 plasmid co-digested by *Sal*I and *Bam*H1: cells were transformed with linearized (for NHEJ) or uncut (for transformation efficiency) plasmid and incubated at 30 °C for 7 days. Values are calculated with respect to transformants of each strain normalized to wild type^4^.

### Heteroallelic recombination assay

The substrate for intrachromosomal recombination contains non-tandem direct repeats of *ade*^**-^ heteroallelles (*ade-L469* and *ade-M375)* separated by a functional *his3*^*+*^ gene^16^. For each assay, single colonies grown on a low adenine plate were inoculated in YEP media for 72 h, cell number was then established and generation numbers calculated for each culture. Cells from each single colony were plated at a density of 5 × 10^4^ per plate onto low adenine media to select for spontaneous *ade*^*+*^ cells. After 6 days growing at 30 °C, the number of recombinants was determined and recombination rate per generation was calculated. The *ade*^*+*^ recombinants were then replicated onto his^-^ plate to determine the proportion of conversion type (*ade*^*+*^ *his*^*+*^) and deletion type (*ade*^*+*^ *his*^**-^). For each strain, the mean recombination frequency was determined from three independent assays. The average recombination frequencies and standard deviation were derived from three independent mean recombination frequencies^49^.

### *C*hromosomal DNA preparation for resection assay

Ten OD_600_ of logarithmically growing overnight culture were collected and the cell wall was digested at 37 °C in CSE buffer (20 mM citrate/phosphate, pH 5.6, 40 mM EDTA, 1.2 M Sorbitol) containing 100 T zymolyase at 1 mg ml^−1^. Spheroplasts were pelleted and lysed in 450 μl of 5 × TE plus 50 μl of 10% SDS followed by treatment with 150 μl of 5 M potassium acetate. The extract was cleared by centrifugation at 20,000*g* for 10 min. Supernatant was transferred to new tube and nucleic acids precipitated using 600 μl isopropanol. The pellet was washed with 70% ethanol. DNA was air dried and resuspended in double distilled water plus 5 μl of 10 mg ml^−1^ RNaseA. After 15 min incubation at 37 °C to allow the digestion of RNA, 4 μl of 10% SDS and 20 μl of 20 mg ml^−1^ proteinase K were added and the solution was incubated overnight at 30 °C. The solution was then extracted twice with equal volume of phenol/cholorform (1:1) and DNA precipitated using isopropanol. The pellet was washed with 70% ethanol, air dried and was resuspended in 100 μl ddH_2_O.

### Analysis of ssDNA processing by *Apo*I digestion

Strains used in resection analysis harbour a single HO cleavage site at the *arg3* locus and a gene coding HO endonuclease under the control of *nmt41* promoter. For induction of HO endonuclease, pre-cultures were grown over-night in the presence of thiamine, washed three times and subsequently cultured in medium in the absence of thiamine. Samples were taken at indicated time points after 14 h (*P*_nmt_ starts transcribing ∼14–16 h following thiamine removal). Resection was measured at 35 bp and 3 kbp from the break based on the location of the *Apo*I restriction sites. After induction of HO endonuclease by removing thiamine, at the indicated times, genomic DNA was extracted from 50 OD_600_ of culture followed by digestion with *Apo*I (Fermentas, #ER1381). The overall rate of resection was assessed by semi-quantitative or real-time quantitative PCR (qPCR)^17^ (35 bp; primer pair 13/14, 3 kbp; primer pair 15/16). Induction of the HO break was assessed by measuring the percentage of uncut DNA using PCR with primer pair 23/24.

### Chromatin immunoprecipitation

Following 15 min cross-linking with formaldehyde at a final concentration of 1%, cells were harvested, washed once in PBS and broken in a Ribolysed with glass beads in genomic DNA extraction buffer. Extracts were sonicated to yield DNA fragment at a size between 500 and 1,000 bp. Rabbit IgG-coated magnetic beads were used to retrieve TAP-tagged proteins. Immune-complexes retrieved using anti-uH2B and anti-H4K20-dimethyl antibodies were enriched by Protein G magnetic Dynabeads. Cross-linking was reversed by incubation at 65 °C for 2 h, DNA was purified using a Gel Extraction Kit (Omega, D2500-02). 35 ng of recovered DNA was used for each quantitative real-time PCR reaction^17^,^32^. For each data point, all reactions were performed in triplicate. The primers used to measure protein enrichment: 0.2 kb, primer pair 17/18; 3 kb, primer pair 15/16; 5.4 kb; primer pair 19/20; 9 kb, primer pair 21/22. Reactions were run on CFX-96 real-time system (Bio-Rad) using Promega GoTaq qPCR Master Mix.

### Transcription analysis by RNA sequencing

Cells we arrested in G2 using the *cdc25.22* allele. *wdr70*^+^ and *wdr70*Δ strains (two biological replicates per condition) were subjected to total RNA extraction and mRNA was purified using poly-T magnetic beads (NEBNext). Sequencing libraries were generated from purified PolyA+ RNA using a NEBNext Ultra RNA Library Prep Kit for Illumina sequencing an Illumina Hiseq2500 platform following the manufacturer's protocol. 125 bp/150 bp paired-end reads were generated. HTSeq v0.6.1 software was used to count the reads and numbers mapped to each gene. Differential expression analysis between two strains was performed using the DESeq R package (1.18.0). Corrected *P*-value of 0.005 and log2 (Fold change) of 1 were set as the threshold for significantly differential expression.

### Human cell culture and analysis

Human cell lines were maintained in culture media supplemented with 10% fetal bovine serum. HEK293T obtained from American Type Culture Collection or their derived cell lines were cultured in DMEM. All cell lines tested negative for mycoplasma. Lentivirus expressing 53BP1shRNA GV248-shTP53BP1 (target sequence: CTTGTTCAGGACAGTCTTT ) was packaged by co-transfecting 293T cells with pLVX-IRES-ZsGreen, pMD.2G and psPAX2 plasmids using X-tremeGENE HP (Roche, 06366244001).

Secreted virus was collected 72 h after transfection. Infectivity of supernatant was determined by positive green fluorescent protein clones in 96-well plates at tenfold serial dilution and cells were infected at a titre of 2 MOI (multiplicity of infection) per cell. Cells with stably integrated lentivirus were propagated in DMEM supplemented with 10% fetal bovine serum up to 1 month for experimental use.

Single duplex siRNA used in this study were purchase from Ribobio. Individual siRNA duplexes used were: DDB1 (siB09531141904, target sequence: 5′- CCUGUUGAUUGCCAAAAAC -3′ or siG1098103838, 5′- CUCCUUGGAGAGACCUCUA -3′), WDR70 (001: siG1117130927, target sequence: 5′- CUGCCAGAAUGGAAGCAUA -3′; 002: siG1117130938, target sequence: 5′- GAUCAAAUGUGGUCAGAGA -3′). Transfections were performed using Lipofectamine 2000 or X-tremeGENE siRNA Transfection Reagent (Roche, 04476093001) following the manufacturer's instruction. Normally, two consecutive rounds of siRNA transfections were carried out and cells were assayed 48 h after second round.

Knockout cell line for WDR70 was obtained by following gRNA provider's protocol (ViewSolid Biotech). Briefly, 293T cells were co-transfected with pCDH-Cas9 and gRNA_WDR70_ plasmids (gRNA targeting sequence for Exon1: 5′- GAGCGCTCTGGGCCCAGCGAAGG -3′). Forty-eight hours after transfection, trypsinized cells were diluted in 96-well plates for amplification and selection of clones. Correct clones were identified by immunoblotting and verified by DNA sequencing for the Exon1 region (primer 40/41).

DSBs were induced by irradiating cells at 1 Gy min^−1^ using a custom-made X-ray machine (Wandong Ltd) and CPT treatment was carried out at a concentration of 2 μM for indicated time. Cells after genotoxic challenge were either fixed by 4% paraformaldehyde or lysed in Lamilli SDS sample buffer for immunofluorescence (IF) or immunoblotting, respectively.

The method for plasmid-based DSB repair system was adapted from a previous report^38^. Briefly, pCMV plasmids (see Supplementary Fig. 6g for details) containing a pair of inverted I-*Sce*I restriction sites were digested thoroughly by I-*Sce*I restriction enzyme, and cutting efficiencies were examined by PCR. Digested plasmids (NHEJ: 5 μg, HR: 10 μg) were transfected into 293T or WDR70 knockout cells and allowed to repair *in vivo*. Forty-eight hours later, recombined or ligated plasmids (HR or NHEJ) were extracted by HighPure PCR Template Preparation Kit (Roche, 11796828001) and subjected to normalization PCR (Primer 35/36). Successfully repaired plasmids were quantified by real-time PCR with appropriate primers specific for HR (Primer 37/38) or NHEJ fragments (Primer 38/39), using Bio-Rad CFX96 Real-Time System. The relative frequency of each repair pathway was defined as fold changes relative to qPCR values of repaired DNA extracted in parallel from wild type or mock-infected 293T cells.

### Statistical analysis

Statistic data are presented as means+standard deviation unless otherwise stated. At least three independent experiments containing parallel controls were performed for each analysis. For quantitative analysis including real-time PCR, survival, repair efficiency and image analysis, three independent experiments were performed unless stated otherwise and each one included three parallel technical repeat samples. Statistical analysis was performed in Microsoft Excel using the Student's *t*-test. All *t*-test assays are two-side tests: *, *P*<0.05; **, *P*<0.01; NS, no significance. Outliers were eliminated by *Q*-test if *Q*_i_>*Q*_(95%)_.

## Additional information

**Accession codes:** The RNA-seq data have been deposited in GEO repository under the accession codes GSE79830.

**How to cite this article:** Zeng, M. *et al*. CRL4^Wdr70^ regulates H2B monoubiquitination and facilitates Exo1-dependent resection. *Nat. Commun.* 7:11364 doi: 10.1038/ncomms11364 (2016).

## Supplementary Material

Supplementary Information Supplementary Figures 1-7 and Supplementary Tables 1-5
