# Supplementary material for: CRL4Wdr70 regulates H2B monoubiquitination and facilitates Exo1-dependent resection
Source: Nat Commun. 2016 Apr 21;7:11364. doi: 10.1038/ncomms11364 (PMC4844679; doi:10.1038/ncomms11364)
Supplement: Supplementary Information — Supplementary Figures 1-7 and Supplementary Tables 1-5 [file ncomms11364-s1.pdf]

Supplementary Figure-1

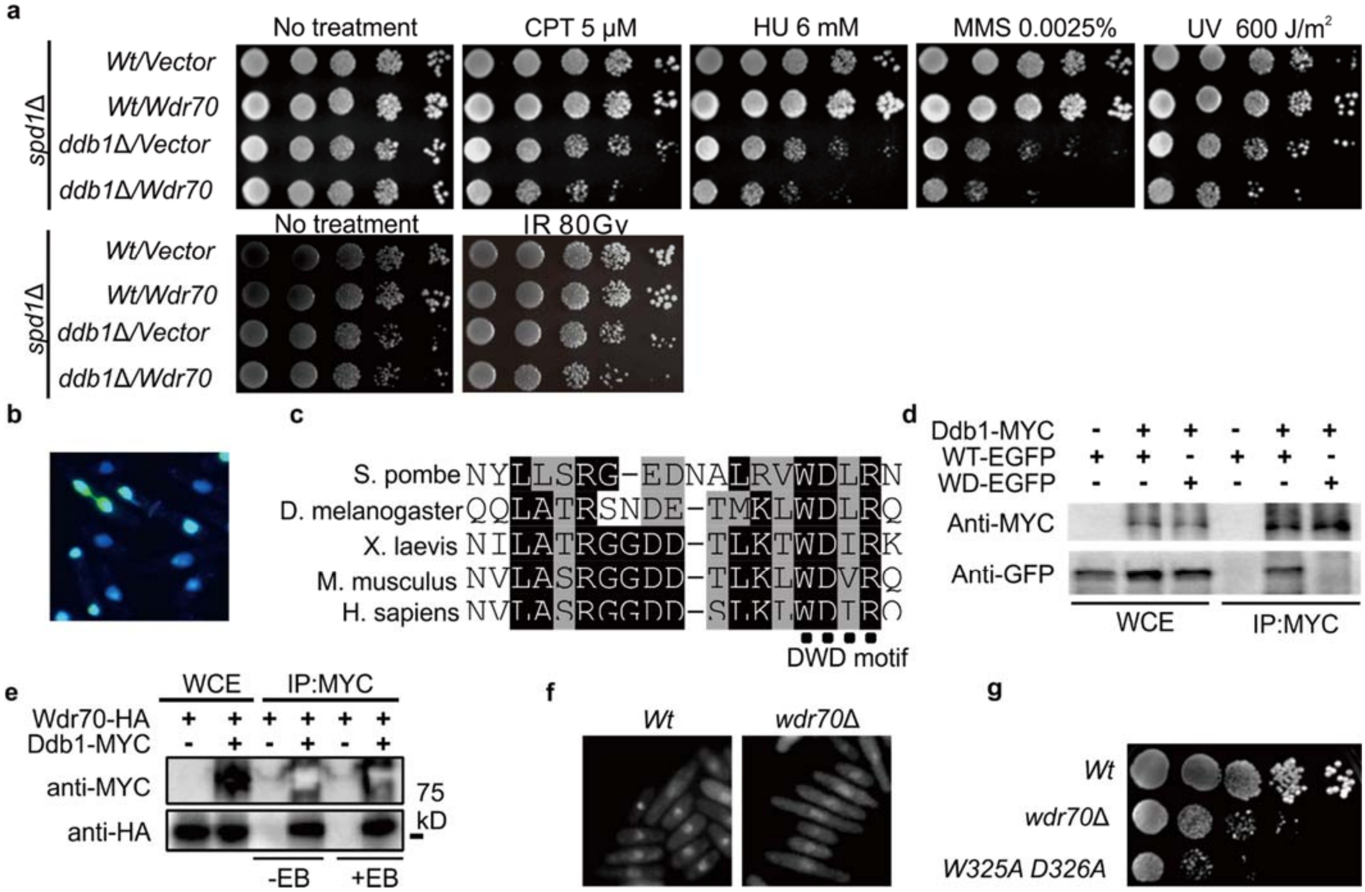

Analysis of Wdr70 as a DCAF protein. (**a**) Top: 10 fold serial dilution of indicated transformed strains onto medium containing the indicated drugs (for UV treatment, cells were plated and immediately irradiated). Bottom: an equivalent experiment using IR. Plates were incubated for 4 days and photographed. Over-expression of *wdr70* from the *nmt41* promoter exacerbates hypersensitivity of *ddb1*Δ to genotoxic challenge but does not exert a similar effect on *ddb1*$^+$ cells. (**b**) An EGFP-tagged Wdr70 (expressed ectopically from an *nmt41* promoter) visualised in methanol-fixed cells by fluorescence microscopy. Nuclei were counterstained with DAPI and an overlaid image is shown. (**c**) Alignment showing conservation of Wdr70 in the indicated eukaryotic species (corresponding to 391 - 409 of the human sequence). Wdr70 contains four clearly identifiable WD40 domains, one of which harbours a variant DWD motif (WDxR, the signature sequence of DCAF protein family). The sixteen residues shown fulfil the following features of DWD motif[1]. The first fourteen residues include three highly conserved residuals, Asp7, Trp13, and Asp14. Four hydrophobic residues (Ile, Leu or Val) are present at positions 1, 10, 12, and 15. Distinct from other DCAF proteins, which have three small residues (Gly, Ser, or Thr) at position 3, 4 and 5, in Wdr70 there is a highly conserved arginine instead of a small residue at position 4 (see Figure 1a). Wdr70 also contains the signature arginine 16 residue following the WD dipeptide for the DWD domain. (**d**) Co-immunoprecipitation experiment for Myc-tagged Ddb1 expressed from its chromosomal locus and Wdr70$^+$ (WT-EGFP) or the WD mutant (WD-EGFP) expressed from an *nmt41* plasmid. (**e**) Co-IP of Myc-tagged Ddb1 and HA-tagged Wdr70 with 10 μg of ethidium bromide (EB) added to the pre-cleared cell lysates and incubated on ice for 10 min before immunoprecipitation with 2 μg of anti-Myc antibody. (**f**) *wdr70*Δ cells visualised during unperturbed logarithmic growth following fixation and staining with DAPI. (**g**) Spot-test analysis. 10-fold serial dilutions of *wdr70*Δ and *wdr70-wd* (*W325A D326A*) to show the growth phenotype. Plates were incubated for 5 days.

Supplementary Figure-2

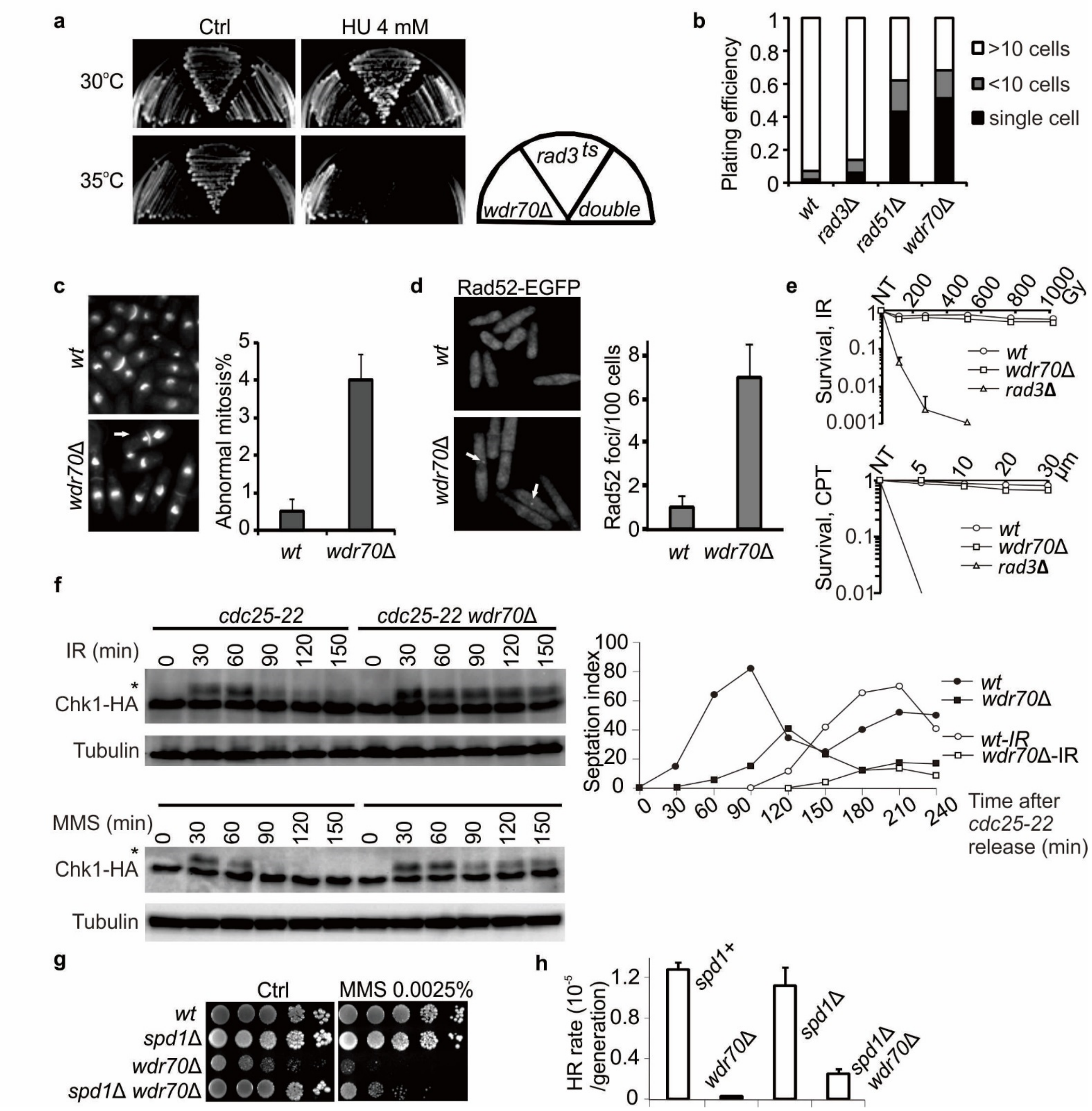

Analysis of *Wdr70* and the DNA damage response. (**a**) The indicated strains were streaked onto rich media agar plates with or without hydroxyurea (HU) and incubated at either 30 or 35°C for 3 days. The double *wdr70*Δ *rad3^{ts}* mutant is lethal in the absence of genotoxic treatment at the non-permissive temperature for *rad3^{ts}* (35°C). The *rad3^{ts}* single mutant only lost viability in the presence of HU. (**b**) Plating efficiency of *wdr70*Δ compared with *rad3*Δ and *rad51*Δ. Single cells were plated onto rich media agar plates and scored microscopically for micro-colony formation. (**c**) Logarithmically growing *wdr70^{+}* (*wt*) and *wdr70*Δ cultures stained with DAPI (DNA) and calcofluor (septum) and scored for the percentage of abnormal mitotic cells. Left: representative images. Arrow marks an abnormal mitosis. (**d**) Spontaneous Rad52 foci visualized in a *rad52-EGFP* background by fluorescence microscopy of unperturbed logarithmically growing cultures. Left: representative images. (**e**) Clonogenic survival analysis for wild type (wt) *wdr70*Δ and *rad3*Δ cells in response to IR or CPT. (**f**) Left: western blot

analysis of IR-induced (40 Gy, top) and MMS-induced (bottom) Chk1 phosphorylation in cultures synchronized in G2 by *cdc22-25* temperature arrest and released after irradiation. Right: mitotic entry of the same IR-treated culture after exposure. The checkpoint activation is more intense and prolonged for *wdr70*Δ cells. This corresponds to a longer checkpoint delay in *wdr70*Δ cells (the time between irradiation and mitotic entry). Asterisk: slow migrating band represents phosphorylated Chk1. (**g**) Spot analysis showing the rescue of the slow growth and MMS hypersensitivity phenotypes of *wdr70*Δ by concomitant *spd1* deletion. Ten-fold series dilution of indicated cells were plated on rich media and cultured in the presence or absence of 0.0025% MMS. (**h**) Analysis of the rates of homologous recombination in the indicated strains. Deletion of *spd1* partially rescues the decrease in spontaneous HR observed in the *wdr70*Δ background.

Supplementary Figure-3

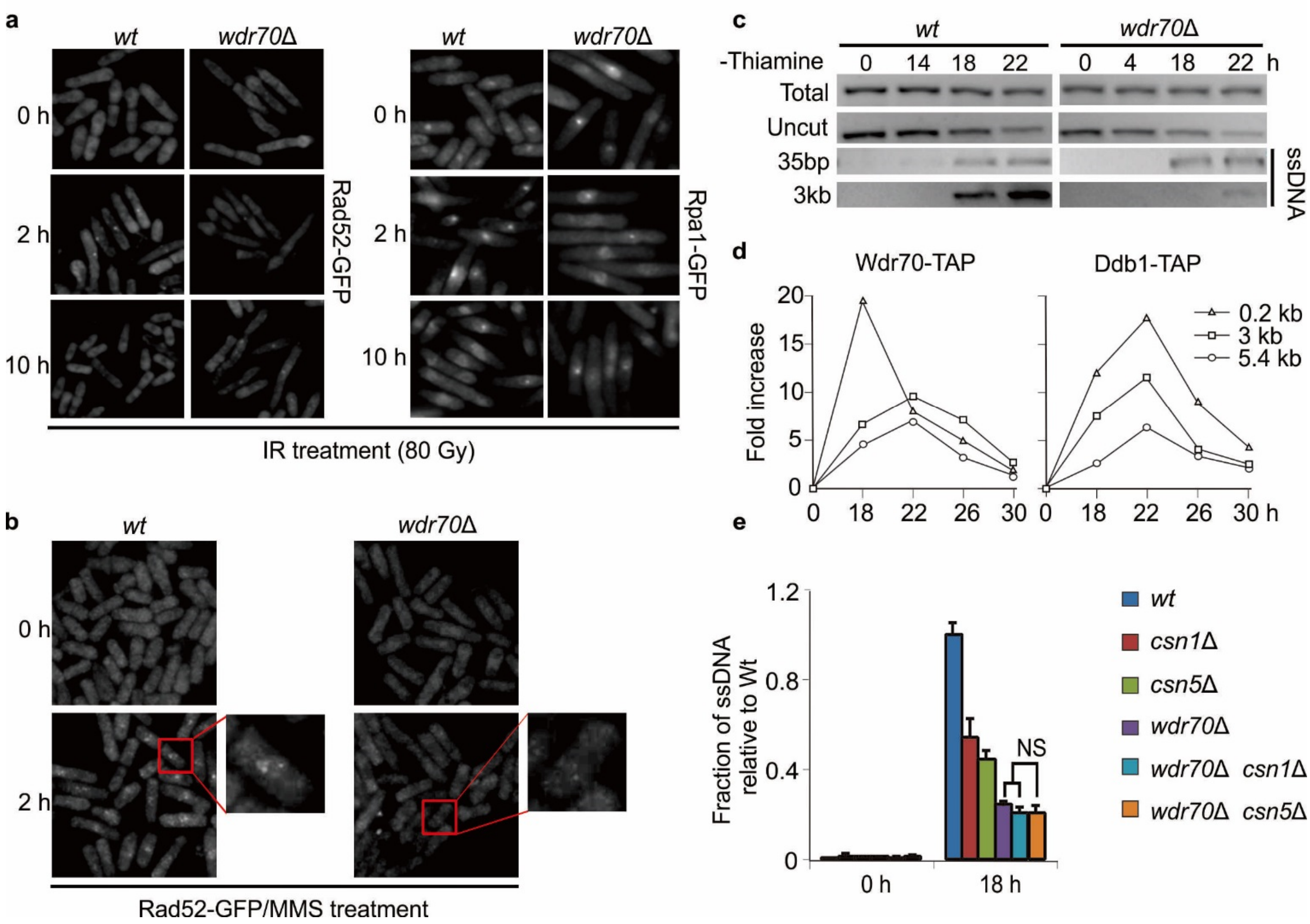

CRL4^{Wdr70} and the response to DNA double strand breaks. All genetic backgrounds are *spd1*Δ. (**a**) Representative fluorescence images of Rad52-GFP and Rpa1-GFP foci at the indicated time points after IR (80 Gy) in *wdr70*$^+$ (wt) and *wdr70*Δ cells. The numbers of foci are quantified in Fig. 2a. (**b**) Analysis of Rad52-GFP foci in *wdr70*$^+$ (wt) and *wdr70*Δ cells in response to MMS treatment. Foci were largely absent in *wdr70*Δ cells (inset). (**c**) Analysis of resection for experiments shown in Fig. 3d,e following an HO-induced DSB after removal of thiamine. PCR was performed across the *Apo*I sites (see Fig 2b) following prior digestion with *Apo*I. Total input genomic DNA was amplified as an internal control. (**d**) ChIP analysis for Wdr70 and Ddb1 chromatin association at the indicated distances from an induced DSB at the indicated times after thiamine removal. Note that the peak of Wdr70 association at 0.2 kb was 18 hour post-induction, 4 hours earlier than Ddb1. (**e**) qPCR quantification of resection in *wdr70*Δ, *csn1*Δ and *csn5*Δ mutants 3 kb from a DSB.

Supplementary Figure-4

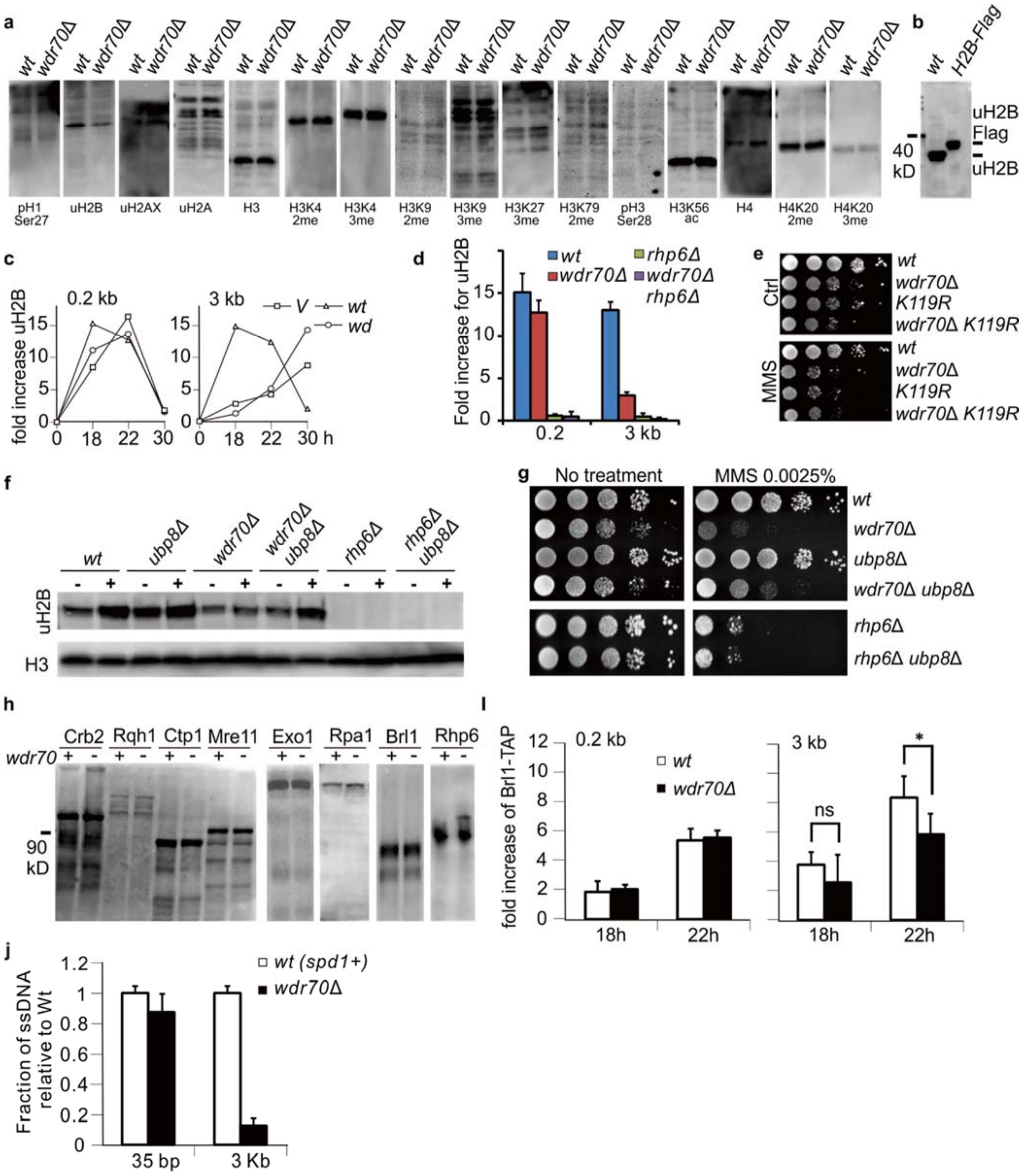

Wdr70-dependent H2B monoubiquitination in response to DSBs. All genetic backgrounds are *spd1*Δ. (**a**) Screen for the indicated modified histone markers by Western blot analysis. Antibodies reported to be specific for each modification shown were used to probe duplicate membranes with extracts *wdr70*+ and *wdr70*Δ run side-by-side. The major reproducible change displayed by *wdr70*Δ cells was a reduced level of a band in the blot probed for with antibody reported to be specific for monoubiquitinated H2B. (**b**) The

specificity of the antibody against uH2B was established by examining the differential migration rate of Flag-tagged and untagged uH2B species during SDS-PAGE (12%) extracted from H2B^{+} (wt) and H2B-*Flag* strains. Thus, the antibody is specific for uH2B. (**c**) Chromatin immunoprecipitation of uH2B to analyse enrichment after DSB formation following induction of the HO endonuclease. A *wdr70*Δ strain was transformed with either an empty *nmt41* vector (V) or plasmids encoding *wdr70*$^{+}$ (wt) or *wdr70-W325A-D326A* (wd). The deficiency of H2B monoubiquitination 3 kb from the DSB break site was complemented by *wdr70*$^{+}$ but not the *wdr70-wd* mutant. (**d**) uH2B enrichment at 0.2 and 3 kb from a DSB assayed by chromatin immunoprecipitation (ChIP) in the indicated backgrounds. uH2B enrichment is dependent on *rhp6*, as expected. (**e**) 10 fold serial dilution of the indicated strains to assay for sensitivity to MMS treatment (0.0025%). The *wdr70*Δ H2B-*K119R* background shows no additive sensitivity when compared to the single *wdr70*Δ and *H2B-K199R* backgrounds. (**f**) Analysis of MMS-induced H2B monoubiquitination in the indicated strains. Note that damage-induced uH2B levels are restored in *wdr70*Δ, but not in *rhp6*Δ, by concomitant deletion of *ubp8*. (**g**) 10 fold serial dilution of the indicated strains to assay for sensitivity to MMS treatment (0.0025%. Note that *ubp8*Δ partially rescued the *wdr70*Δ (upper panel) but not the *rhp6*Δ (lower panel) hypersensitivity to MMS. (**h**) Immunoblotting of cell lysates from the indicated TAP-tagged strains in *wdr70*$^{+}$ (+) and *wrd70*Δ (-) backgrounds. (**i**) Recruitment of Brl1-TAP 0.2 and 3 kb distal to the HO site in the *wdr70*Δ cells assayed by ChIP and quantitative PCR. (**j**) In the presence of *spd1*$^{+}$, loss of *wdr70* results in a similar defect in resection as seen in *spd1* null cells (c.f. Fig. 4c). n = 3 biological repeats. Error bars = s.d. * = $p<0.05$, t-test.

Supplementary Figure-5

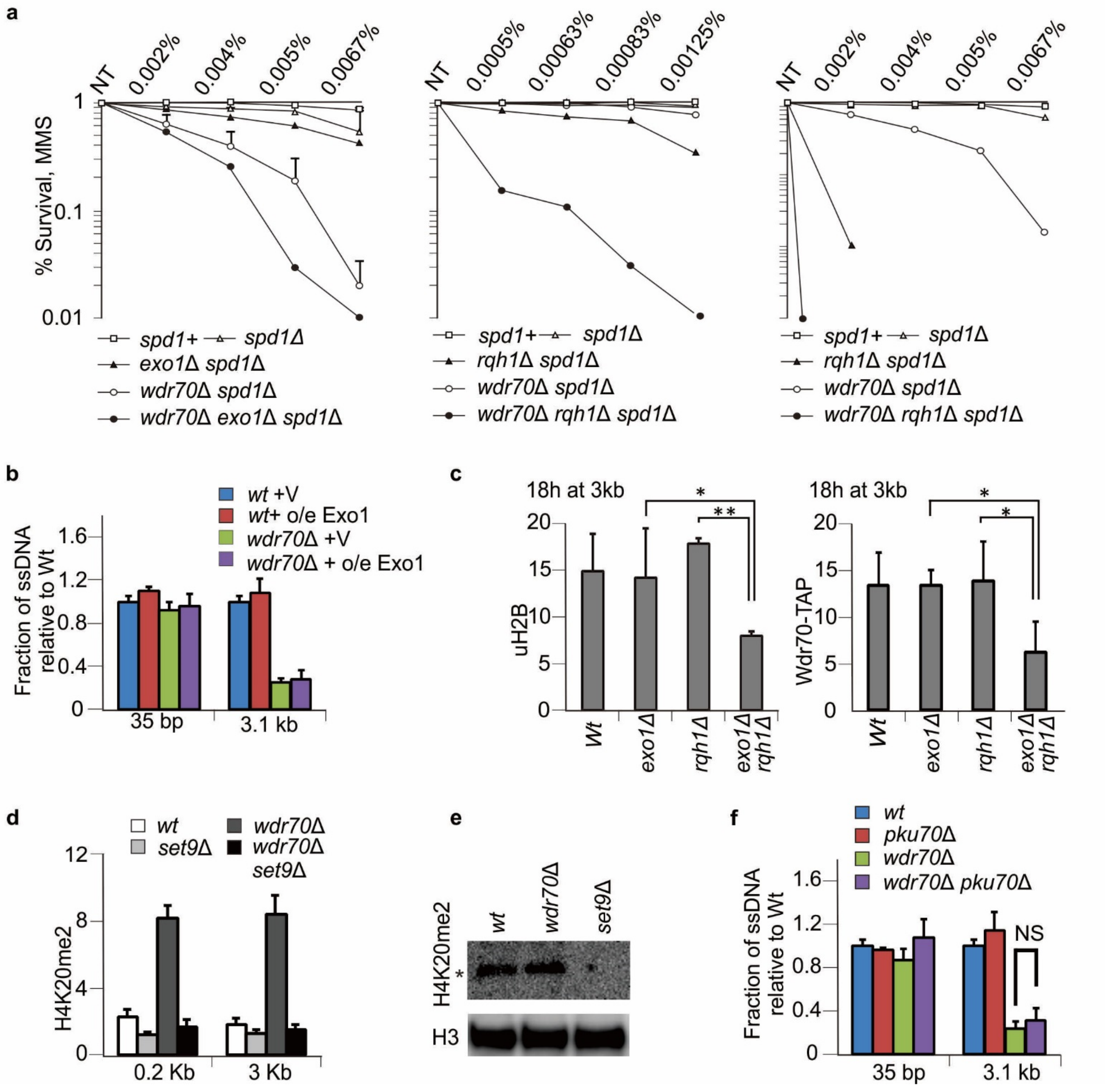

Interplay between Wdr70 and resection factors. All genetic backgrounds are *spd1*Δ unless annotated *spd1*⁺ (**a**) MMS clonogenic survival analysis of *wdr70*Δ combined with *exo1*Δ or *rqh1*Δ. *wdr70*Δ displayed a synergistic sensitivity with *rqh1*Δ at low (0.0005%-0.00125%) and high doses (0.002% - 0.0067%) of MMS treatment. In contrast, *exo1*Δ *wdr70*Δ showed an apparently additive sensitivity. (**b**) Analysis of ssDNA formation at the *Apo*I site 3kb from the HO break site in the indicated strains. Exo1 overexpression does not rescue the resection defect of the *wdr70*Δ mutant. (**c**) H2B monoubiquitination and Wdr70 loading analysed by Chromatin immunoprecipitation (ChIP) in the *exo1*Δ, *rqh1*Δ and *exo1*Δ *rqh1*Δ backgrounds. When long-range resection is disrupted by concomitant deletion of the *exo1* and *rqh1* genes H2B modification levels and Wdr70 recruitment were reduced. n = 3 biological repeats. Error bars = s.d. * = p<0.05,** = p<0.01, t-test. (**d**) ChIP analysis of H4K20me2 in *wdr70*Δ, *set9*Δ and *wdr70*Δ *set9*Δ background. Elevated H4K20me2 in *wdr70*Δ was reduced to wild type level by loss of *set9* function. (**e**) Western blot analysis of the total level of H4K20me2 in *wdr70*⁺, *wdr70*Δ and *set9*Δ. Deletion

of *set9* abolishes H4K20 di-methylation. (**f**) Analysis of ssDNA formation at the *Apo*I site 3kb from the HO break site in the indicated strains. pKu70 loss does not rescue the resection defect of *wdr70*Δ.

Supplementary Figure-6

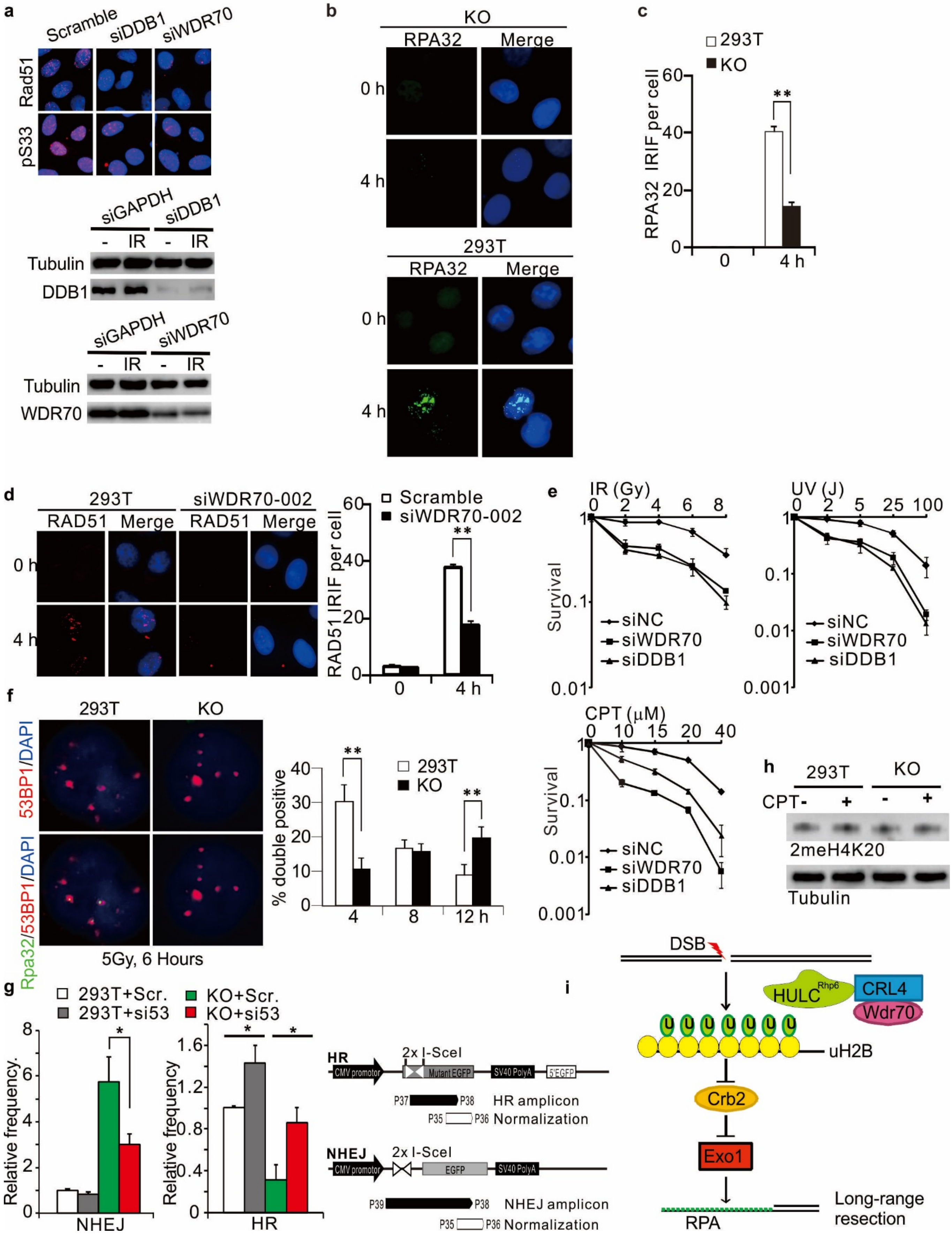

Conserved roles of mammalian CRL4^{WDR70} in regulating homologous recombination. (**a**) Assessment of foci for Rad51 and phospho-Rpa32 (pS33) by indirect immunofluorescence in 293T cells transfected with DDB1 or WDR70 siRNA (top). Knockdown efficiency of each gene was evaluated by immunoblotting (bottom). (**b**) Examples of IR-induced foci for Rpa32 in CRISPR knockout WDR70 cells. (**c**) Quantification of IR-induced foci for Rpa32 in CRISPR knockout WDR70 cells. n = 3 biological repeats. Error bars = s.d. ** = p<0.01, t-test. (**d**) A second siRNA against WDR70 was used to establish the specificity of knockdown. Rad51 foci following IR were reduced to a similar extent to that reported in Fig.1a. n = 3 biological repeats. Error bars = s.d. ** = p<0.01, t-test. (**e**) Survival analysis of siRNA treated cells exposed to the indicated doses of genotoxin. (**f**). Immunostaining (left) and quantification (right) of pS33-positive (green) 53BP1 foci (red) for 293T and WDR70 knockout (KO) cells. Note that in 293T cells, the percentage of double positive foci decreased after 4 hours post-IR whilst the foci number elevated until 12 hours in KO cells. Nuclei were counterstained by DAPI. n = 3 biological repeats. Error bars = s.d. ** = p<0.01, t-test. (**g**) Left: quantification of repair efficiency for I-*Sce*I-induced DSB (c.f. Fig. 6d) for WDR70 knockout (KO) and parental 293T cells following transfection with si53BP1. n = 3 biological repeats. Error bars = s.d. * = P<0.05, t-test. Right: diagrammatic representation of the constructs used. (**h**) CRISPR knockout of WDR70 does not affect global Me2H4K20 levels. (**i**) Model for CRL4^{Wdr70}-mediated histone modification and long-range resection after DSB induction. HULC enzyme together with CRL4^{Wdr70} catalyse H2B monoubiquitination, which triggers the removal of chromatin barrier formed by Crb2^{53BP1} and stimulates Exo1 recruitment, long-range resection and HR . In mammalian cells, loss of this mechanism results in persistent occupancy of 53BP1 on DSBs, therefore favours NHEJ but blocks resection required for HR pathway.

Supplementary Figure-7

Figure 1b

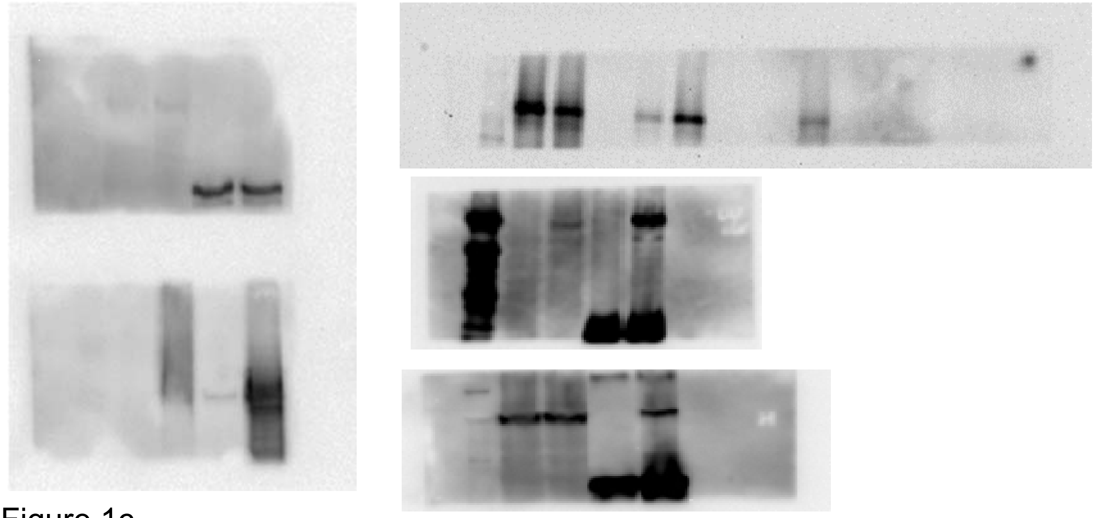

Figure 1c

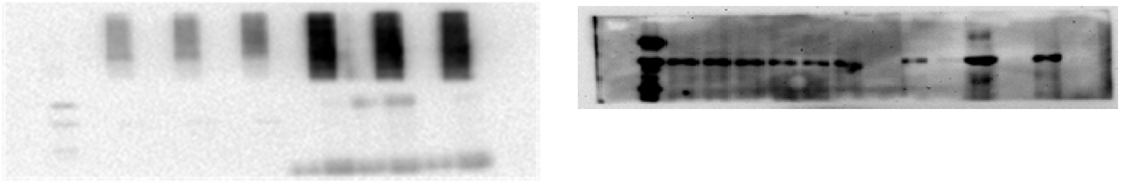

Figure 2b

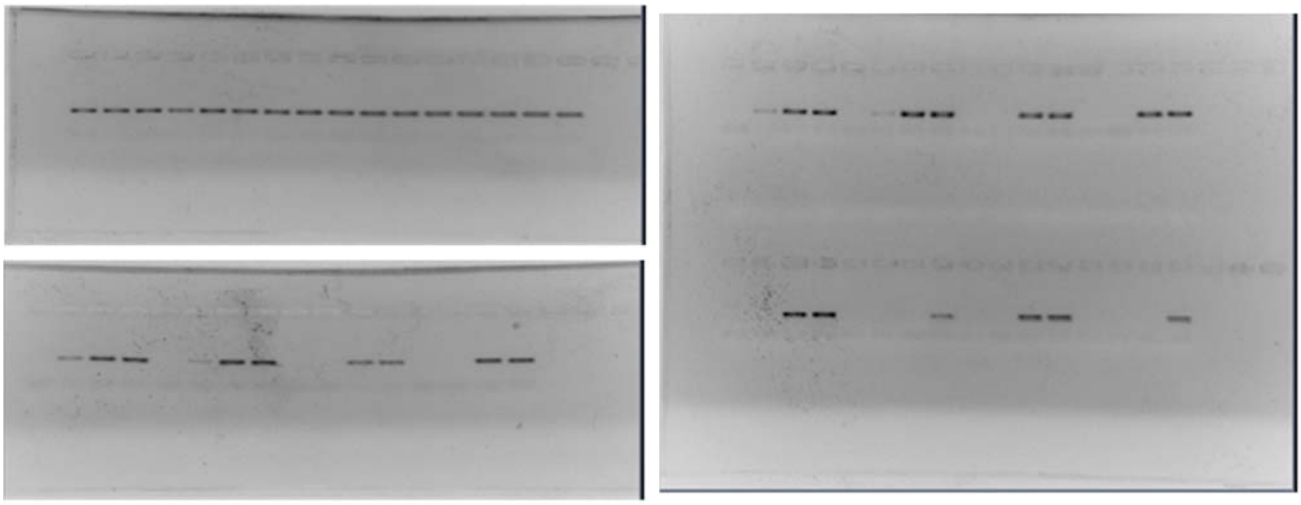

Figure 3a

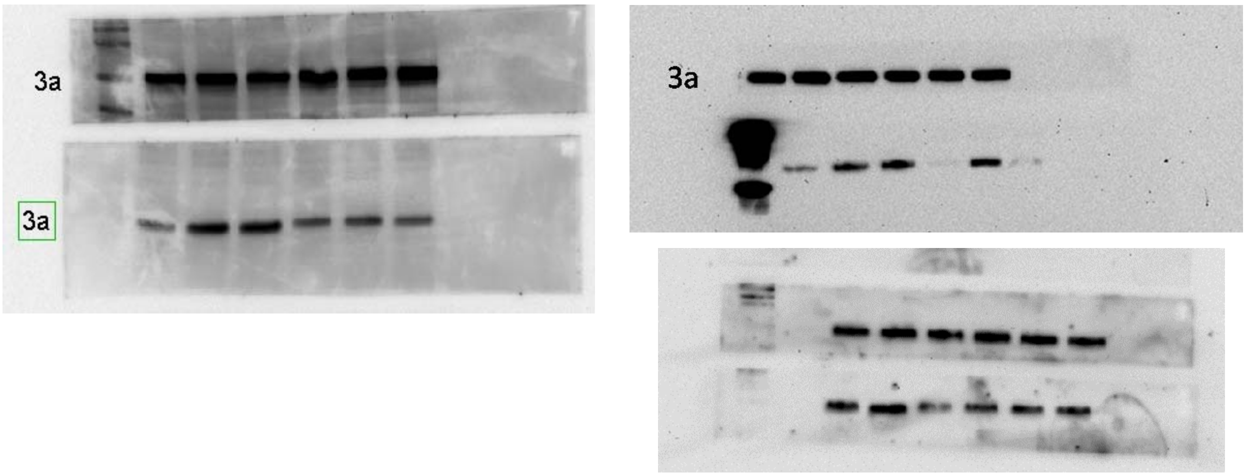

Figure 3c

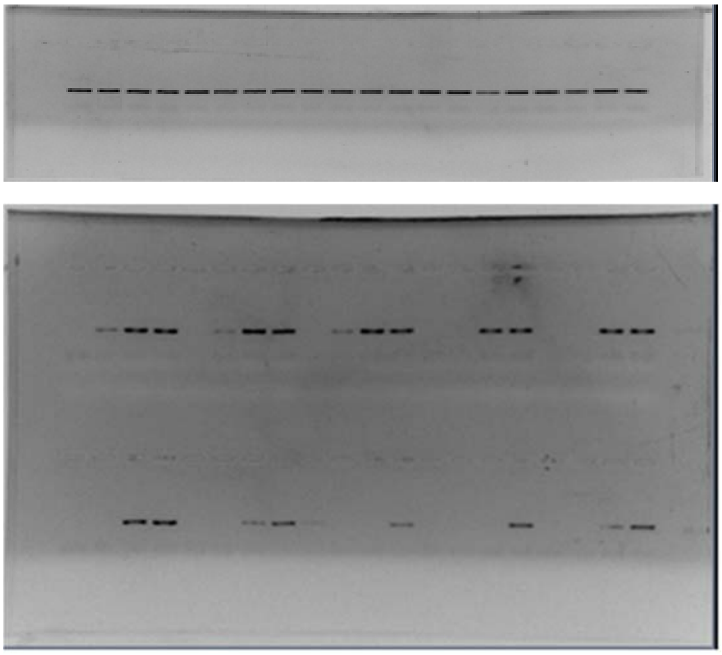

Figure 3f

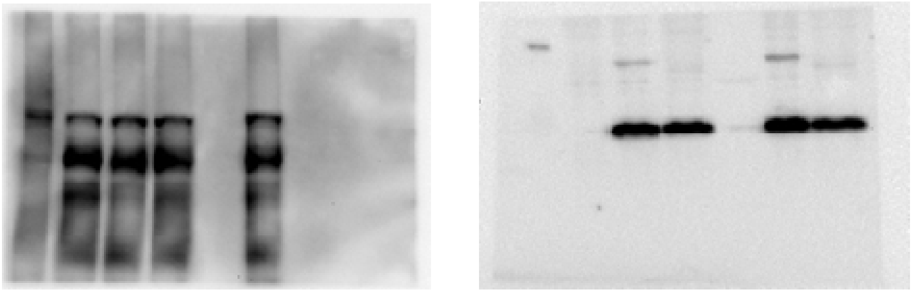

Figure 3h

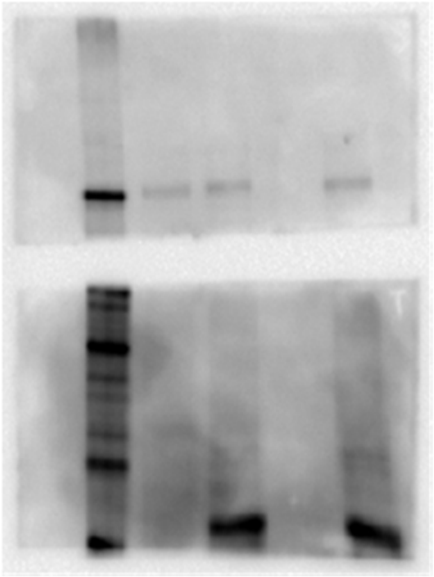

Figure 6c

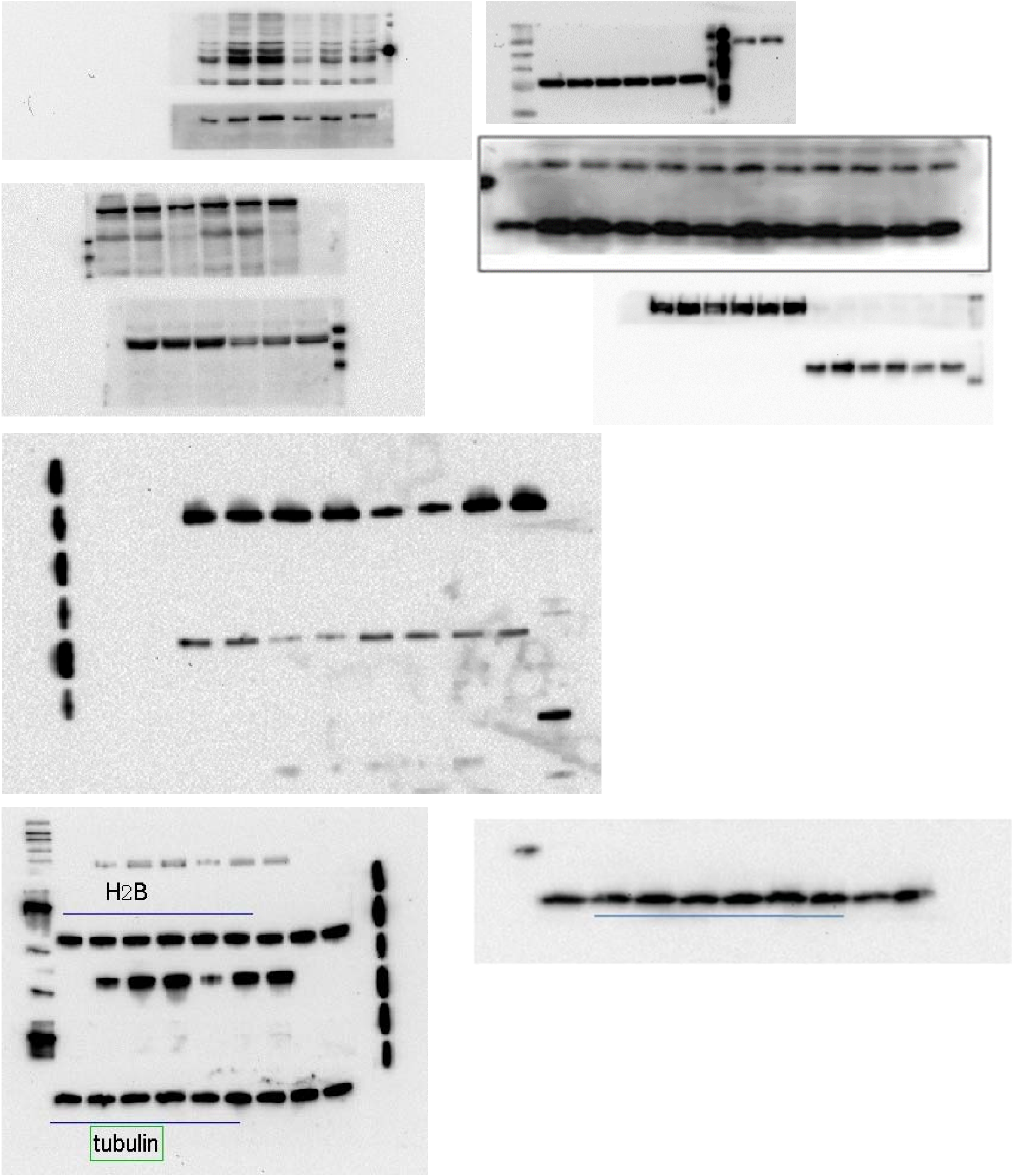

Figure 6d

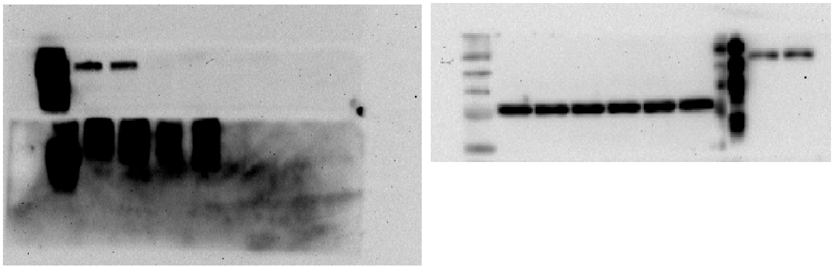

Figure 6g

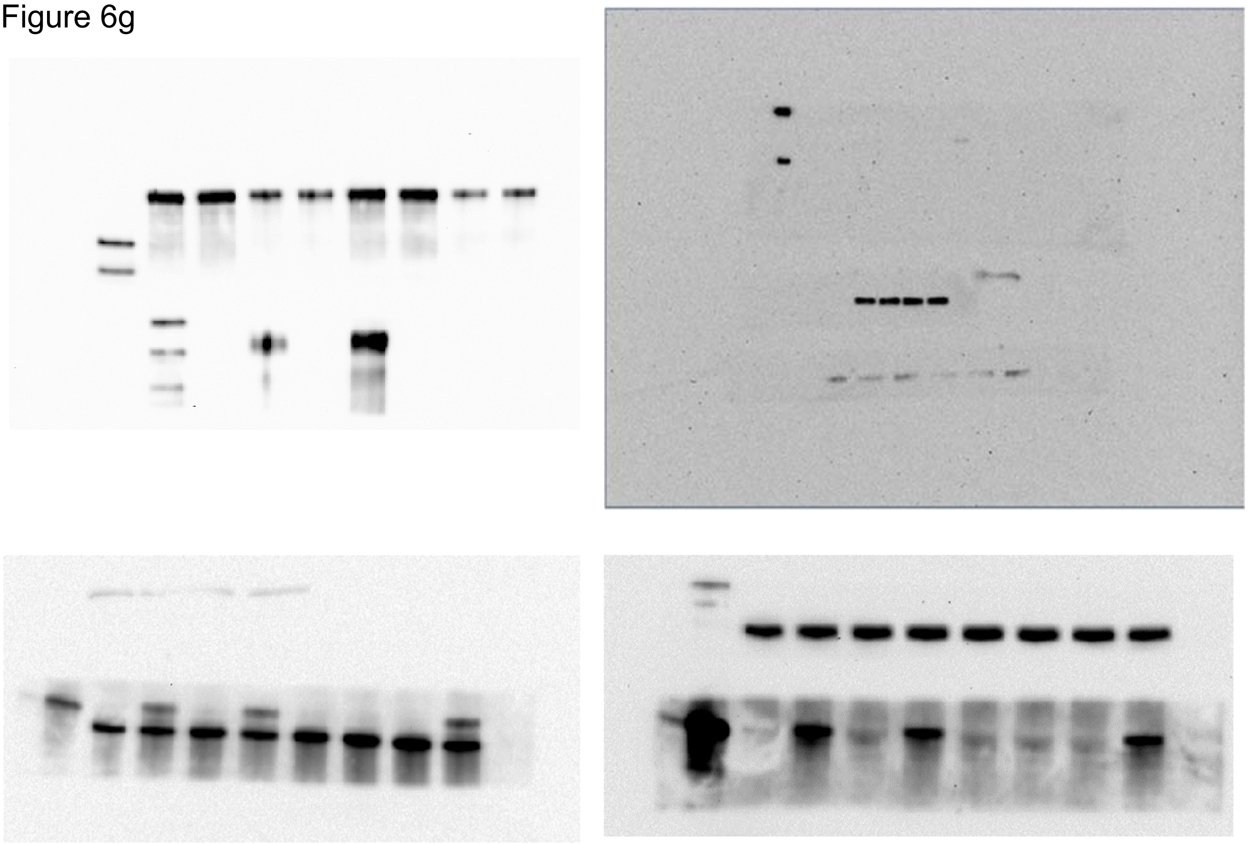

The uncropped blots used in the main figures.

Supplementary Table 1. Transcripts differing between *wdr70*$^{+}$ and *wdr70*Δ

Gene_id	count_Sp2_wdr70	count_Sp1_Wt	log2.change.	p value	q value	Gene Name
SPAC1002.19	253.3749672	49.25398893	2.363	8.85E-35	1.30E-32	urg1
SPAC1006.01	1233.15162	593.9338431	1.054	9.61E-53	2.19E-50	psp3
SPAC1006.03c	414.4168503	173.73575	1.2542	2.87E-24	2.52E-22	red1
SPAC1039.09	89.62801332	42.52004513	1.0758	2.82E-05	0.0004206	isp5
SPAC11D3.09	136.3355696	34.63171097	1.977	6.49E-16	3.53E-14	SPAC11D3.09
SPAC11H11.02c	137.778274	43.4820371	1.6639	5.01E-13	2.01E-11	mug162
SPAC11H11.03c	122.8102154	52.52476163	1.2254	5.52E-08	1.38E-06	SPAC11H11.03c
SPAC139.05	19.29617188	3.078374308	2.6481	0.00029574	0.0035173	SPAC139.05
SPAC15F9.01c	34.08389239	9.04272453	1.9143	7.65E-05	0.0010279	SPAC15F9.01c
SPAC16C9.05	80.79144862	212.9850224	-1.3985	6.92E-15	3.29E-13	cph1
SPAC16E8.16	494.3066086	213.5622176	1.2107	3.41E-27	3.32E-25	sua7
SPAC17A5.07c	286.9178454	105.0495233	1.4496	3.83E-21	2.74E-19	ulp2
SPAC17D4.01	663.2833661	1359.871851	-1.0358	4.94E-54	1.33E-51	pex7
SPAC186.02c	1472.820895	120.2489964	3.6145	3.72E-291	8.48E-288	SPAC186.02c
SPAC186.03	52.29803594	20.58662819	1.345	0.00013957	0.0017609	SPAC186.03
SPAC186.07c	102.7926913	49.44638732	1.0558	9.98E-06	0.00017208	SPAC186.07c
SPAC186.09	452.828856	120.4413948	1.9106	5.22E-47	9.93E-45	SPAC186.09
SPAC1F8.01	104.956748	46.17561462	1.1846	9.98E-07	2.12E-05	ght3
SPAC1F8.02c	345.3473753	54.83354236	2.6549	4.64E-53	1.12E-50	shu1
SPAC20H4.11c	118.8427782	57.14232309	1.0564	2.01E-06	4.01E-05	rho5
SPAC212.08c	14.4270444	43.86683389	-1.6044	9.02E-05	0.0011945	SPAC212.08c
SPAC222.07c	54.64243065	112.3606622	-1.04	8.11E-06	0.00014141	hri2
SPAC222.15	151.1232901	56.18033112	1.4276	1.28E-11	4.38E-10	meu13
SPAC22F3.12c	44.72383763	133.5244856	-1.578	1.49E-11	5.03E-10	rgs1
SPAC22G7.04	282.5897321	138.5268439	1.0285	7.41E-13	2.87E-11	pan2
SPAC23A1.20	15.68941078	44.44402907	-1.5022	0.00017379	0.0021568	new11
SPAC23H3.13c	62.93798118	138.1420471	-1.1341	1.08E-07	2.66E-06	gpa2
SPAC24C9.07c	389.7105368	133.5244856	1.5453	9.71E-31	1.20E-28	bgs2
SPAC26H5.06	77.00434947	166.424611	-1.1119	9.58E-09	2.65E-07	pot1
SPAC27D7.12c	158.5171503	55.02594076	1.5265	3.20E-13	1.32E-11	but1
SPAC27F1.05c	29.57544101	4.617561462	2.6792	6.53E-06	0.00011694	SPAC27F1.05c
SPAC29B12.08	224.1602023	527.556397	-1.2348	1.01E-28	1.12E-26	clr5
SPAC32A11.01	333.9860778	92.54362764	1.8516	9.42E-34	1.30E-31	mug8
SPAC32A11.02c	457.8783215	207.9826642	1.1385	4.00E-23	3.21E-21	SPAC32A11.02c
SPAC343.17c	4.508451374	153.9187154	-5.0934	2.02E-37	3.17E-35	SPAC343.17c
SPAC343.21	139.2209784	37.51768688	1.8917	2.17E-15	1.10E-13	SPAC343.21
SPAC4G9.07	90.52970359	42.90484192	1.0773	2.52E-05	0.00038451	mug133
SPAC513.03	19.83718605	55.21833915	-1.4769	3.60E-05	0.00052494	mfm2
SPAC688.13	83.13584334	177.1989211	-1.0918	5.43E-09	1.54E-07	scn3
SPAC6C3.04	532.3579382	1078.970195	-1.0192	3.96E-42	7.23E-40	cit1
SPAC750.08c	1.80338055	20.2018314	-3.4857	2.87E-05	0.00042726	SPAC750.08c
SPAC869.03c	110.1865516	45.59841944	1.2729	1.15E-07	2.83E-06	SPAC869.03c
SPAC869.06c	369.1519985	6.34914701	5.8615	3.05E-89	1.16E-86	SPAC869.06c
SPAC869.07c	207.9297774	53.10195681	1.9693	2.45E-23	2.04E-21	mel1
SPAC869.08	110.5472277	30.59134469	1.8535	3.14E-12	1.14E-10	pcm2
SPAC869.09	23.26360909	3.270772702	2.8304	3.89E-05	0.000562	SPAC869.09
SPAC8E11.10	250.8502345	804.225288	-1.6808	2.25E-67	7.90E-65	SPAC8E11.10
SPAC977.15	140.4833448	43.28963871	1.6983	1.35E-13	5.82E-12	SPAC977.15
SPAC977.16c	420.3680061	137.7572503	1.6095	6.46E-35	9.84E-33	dak2
SPAC977.17	194.7650994	72.91899142	1.4174	2.07E-14	9.36E-13	SPAC977.17
SPAP8A3.04c	5000.774264	1816.43324	1.461	0	0	hsp9
SPAPB1A11.02	115.0556791	57.33472149	1.0049	7.12E-06	0.0001256	SPAPB1A11.02
SPAPJ695.01c	53.74074038	17.70065227	1.6022	1.12E-05	0.0001865	SPAPJ695.01c
SPBC11C11.06c	695.563878	333.4264172	1.0608	8.54E-31	1.08E-28	SPBC11C11.06c
SPBC1348.12	181.2397452	66.9546412	1.4366	9.30E-14	4.05E-12	SPBC1348.12
SPBC1348.14c	320.8213998	95.04480676	1.7551	2.54E-30	3.05E-28	ght7
SPBC1921.04c	201.9786216	61.56748616	1.714	4.20E-19	2.70E-17	SPBC1921.04c
SPBC1D7.05	56.26547315	172.9661564	-1.6202	4.83E-15	2.35E-13	byr2
SPBC21C3.08c	1075.896836	2681.648819	-1.3176	1.25E-153	1.42E-150	car2
SPBC23G7.12c	551.6541101	271.2817359	1.024	1.77E-23	1.50E-21	rpt6
SPBC23G7.15c	2189.303987	950.640466	1.2035	1.88E-113	1.43E-110	rpp202
SPBC27B12.03c	512.1600761	248.1939286	1.0451	1.35E-22	1.07E-20	erg32
SPBC29B5.02c	53.19972621	152.9567234	-1.5236	1.94E-12	7.25E-11	isp4
SPBC2A9.02	51.57668372	108.8974911	-1.0782	6.02E-06	0.00010863	SPBC2A9.02
SPBC32H8.11	110.5472277	24.43459607	2.1777	1.03E-14	4.74E-13	mei4
SPBC336.02	221.6354695	103.7027345	1.0957	2.34E-11	7.80E-10	SPBC336.02
SPBC359.04c	71.59420782	178.7381083	-1.3199	9.06E-12	3.18E-10	pfl7
SPBC4B4.06	89.98868943	33.28492221	1.4349	1.56E-07	3.80E-06	vps25
SPBC56F2.06	49.59296511	20.2018314	1.2956	0.00031242	0.0036775	mug147
SPBC839.11c	24.88665158	57.91191667	-1.2185	0.0002676	0.0032247	hut1
SPBC947.05c	120.1051446	39.24927243	1.6136	4.10E-11	1.35E-09	frp2
SPBCPT2R1.08c	79.16840613	12.50589563	2.6623	1.96E-13	8.36E-12	tlh2

SPBP19A11.02c	37.69065349	81.38452077	-1.1105	6.11E-05	0.0008426	SPBP19A11.02c
SPBP4G3.02	534.702333	1858.183692	-1.7971	3.38E-168	5.14E-165	pho1
SPBP4G3.03	67.98744672	29.82175111	1.1889	7.87E-05	0.0010505	fub2
SPBPB21E7.04c	1571.285473	622.6012038	1.3356	7.14E-96	3.62E-93	SPBPB21E7.04c
SPBPB21E7.11	668.8738459	273.782915	1.2887	1.05E-39	1.77E-37	SPBPB21E7.11
SPBPB2B2.05	82.59482917	191.0516055	-1.2098	4.62E-11	1.51E-09	SPBPB2B2.05
SPBPB2B2.06c	164.10763	408.461791	-1.3156	8.23E-25	7.52E-23	SPBPB2B2.06c
SPBPB2B2.18	94.13646469	189.7048167	-1.0109	1.49E-08	4.01E-07	SPBPB2B2.18
SPBPB8B6.02c	290.1639304	74.45817858	1.9624	8.88E-32	1.16E-29	SPBPB8B6.02c
SPCC11E10.01	194.9454374	479.4567985	-1.2983	2.77E-28	2.94E-26	SPCC11E10.01
SPCC1620.03	96.30052135	44.05923228	1.1281	6.53E-06	0.00011694	mug163
SPCC16A11.06c	52.47837399	106.9735072	-1.0275	1.63E-05	0.00026159	gpi10
SPCC1827.04	680.5958194	230.1084795	1.5645	2.59E-53	6.57E-51	vms1
SPCC18B5.11c	140.1226687	68.49382835	1.0326	4.06E-07	9.09E-06	cds1
SPCC330.03c	19.47650994	65.22305565	-1.7436	4.01E-07	9.03E-06	SPCC330.03c
SPCC330.05c	123.8922438	0	7.9529	6.53E-28	6.78E-26	ura4
SPCC338.12	440.5658683	215.8709984	1.0292	3.26E-19	2.13E-17	pbi2
SPCC569.09	843.0804069	354.5902406	1.2495	2.26E-47	4.49E-45	SPCC569.09
SPCC63.10c	198.7325366	84.27049668	1.2377	3.25E-12	1.17E-10	sec59
SPCC737.03c	706.2038232	321.4977168	1.1353	1.26E-34	1.80E-32	ima1
SPCC737.04	1391.127756	467.3356996	1.5737	5.80E-108	3.79E-105	SPCC737.04
SPMTR.01	37.69065349	0	6.2361	1.77E-10	5.65E-09	mat2-Pc
SPNCRNA.1018	64.20034757	25.39658804	1.3379	2.63E-05	0.0003979	-//-
SPNCRNA.103	551.6541101	121.5957852	2.1817	4.51E-67	1.47E-64	-//-
SPNCRNA.1042	192.4207046	78.69094325	1.29	1.47E-12	5.56E-11	-//-
SPNCRNA.1115	135.0732032	290.7139737	-1.1059	4.33E-14	1.92E-12	-//-
SPNCRNA.1120	69.97116532	33.4773206	1.0636	0.00024617	0.0029822	-//-
SPNCRNA.1299	267.0806594	125.4437531	1.0902	2.73E-13	1.14E-11	-//-
SPNCRNA.1304	111.9899321	28.85975914	1.9562	3.53E-13	1.44E-11	-//-
SPNCRNA.1366	265.818293	99.08517304	1.4237	3.17E-19	2.10E-17	-//-
SPNCRNA.1394	168.9767575	52.71716003	1.6805	7.79E-16	4.04E-14	-//-
SPNCRNA.1487	56.62614926	18.08544906	1.6466	4.27E-06	8.06E-05	-//-
SPNCRNA.1511	218.0287084	85.80968384	1.3453	7.33E-15	3.45E-13	-//-
SPNCRNA.1527	206.4870729	56.9499247	1.8583	1.37E-21	9.93E-20	-//-
SPNCRNA.1553	123.7119057	49.44638732	1.323	7.24E-09	2.03E-07	-//-
SPNCRNA.1595	73.93860253	34.63171097	1.0942	0.00011543	0.0014808	-//-
SPNCRNA.1604	31.91983573	6.156748616	2.3742	1.20E-05	0.00019745	-//-
SPNCRNA.279	60.0525723	22.70301052	1.4033	2.51E-05	0.00038423	-//-
SPNCRNA.471	55.00310676	18.66264424	1.5594	1.31E-05	0.00021451	-//-
SPNCRNA.717	27.77206046	5.387155039	2.366	4.59E-05	0.00065273	-//-
SPNCRNA.814	102.6123533	47.90720017	1.0989	5.17E-06	9.45E-05	-//-
SPNCRNA.828	64.38068562	26.35858001	1.2884	4.32E-05	0.00061901	-//-
SPNCRNA.901	90.71004165	37.32528849	1.2811	1.34E-06	2.76E-05	-//-
SPNCRNA.906	1892.828225	861.5600095	1.1355	6.73E-90	2.79E-87	-//-
SPNCRNA.942	91.97240803	35.40130454	1.3774	2.71E-07	6.34E-06	-//-
SPNCRNA.944	42.19910486	10.77431008	1.9696	7.32E-06	0.00012865	-//-
SPNCRNA.989	92.15274609	37.71008527	1.2891	9.84E-07	2.10E-05	-//-

Differentially expressed genes between *wdr70*$^{+}$ and *wdr70*Δ in G2 (cdc25-22) arrested cells. Read counts and statistics from RNAseq are shown for genes above a threshold change of log2 > 1.

Supplementary Table 2. List of strains used in this study

Strain	Genotype
**TC501**	*h- ade6-704 leu1-32 ura4D-18 his3-D1*
**TC503**	*h+ ade6-704 leu1-32 ura4D-18 his3-D1*
**SP526**	*h- ade6-704 leu1-32 ura4D-18 his3-D1 wdr70-3HA::kanMX6*
**SP533**	*h- ade6-704 leu1-32 ura4D-18 his3-D1 wdr70-TAP::kanMX6*
**SP562**	*h+ ura4D-18 his3-D1 ddb1-13myc::kanMX6*
**SP1114**	*h- ade6-704 leu1-32 ura4D-18 his3-D1 ddb1-TAP::kanMX6*
**SP1111**	*h- ade6-704 leu1-32 ura4D-18 his3-D1 exo1-TAP::kanMX6*
**SP1302**	*h- ade6-704 leu1-32 ura4D-18 his3-D1 mre11-TAP::kanMX6*
**SP1113**	*h- ade6-704 leu1-32 ura4D-18 his3-D1 ctp1-TAP::kanMX6*
**SP1013**	*h- ade6-704 leu1-32 ura4D-18 his3-D1 rhp6-TAP::kanMX6*
**LD412**	*h- ura4-D18 leu1-32::TAP-Crb2(leu1+) crb2D::ura4+ his3-D1*
**SP896**	*h+ htb1-3FLAG::kanMX6* (gifted by Dr S. S. Grewal)
**SP897**	*h+ htb1-K119R-3FLAG::kanMX6* (gifted by Dr S. S. Grewal)
**SP635**	*h+ ade6-704 leu1-32 ura4D-18 his3-D1 wdr70::ura4+*
**SP636**	*h- ade6-704 leu1-32 ura4D-18 his3-D1 wdr70::ura4+*
**SP1102**	*h- ade6-704 leu1-32 ura4D-18 his3-D1 wdr70-3HA::kanMX6 ddb1-13myc::kanMX6*
**SP642**	*h- ade6-704 leu1-32 ura4D-18 his3-D1 cdc25-22*
**SP902**	*h+ ade6-704 leu1-32 ura4D-18 his3-D1 cdc25-22 wdr70::ura4+*
**SP876**	*h- ade6-704 leu1-32 ura4D-18 his3-D1 wdr70::kanMX6*
**SP628**	*h- ade6-704 leu1-32 ura4D-18 his3-D1 ddb1::kanMX6*
**SP815**	*h+ ade6-704 leu1-32 ura4D-18 his3-D1 spd1::hphMX6*
**SP792**	*h+ leu1-32 ura4D-18 his3-D1 rad52-YFP::kanMX6*
**SP831**	*h+ leu1-32 ura4D-18 rad52-YFP::kanMX6 wdr70::ura4+*
**DY645**	*h+ leu1-32 his3-D1 rad11(rpa1)-YFP(leu1+)*
**SP866**	*h+ leu1-32 his3-D1 rad11(rpa1)-YFP(leu1+) wdr70::ura4+*
**SP878**	*h- leu1-32 ura4D-18 his3-D1 exo1:ura4+*
**SP879**	*h+ leu1-32 ura4D-18 his3-D1 ctp1::kanMX6*
**SP880**	*h- leu1-32 ura4D-18 his3-D1 mre11::kanMX6*
**SP959**	*h+ leu1-32 ura4D-18 his3-D1 wdr70:kanMX6 spd1::hphMX6*
**TMN3301**	*h- leu1-32 ura4D-18 his3-D1 rqh1::ura4+*
**SP1136**	*h+ leu1-32 ura4D-18 his3-D1 exo1:ura4+ spd1::hphMX6*
**SP1138**	*h+ leu1-32 ura4D-18 his3-D1 exo1:ura4+ wdr70::kanMX6 spd1::hphMX6*
**SP1142**	*h+ leu1-32 ura4D-18 his3-D1 ctp1::kanMX6 spd1::hphMX6*
**SP1143**	*h+leu1-32 ura4D-18 his3-D1 ctp1::kanMX6 wdr70::ura4+ spd1::hphMX6*
**SP1131**	*h+ leu1-32 ura4D-18 his3-D1 rqh1::ura4+ wdr70::kanMX6 spd1::hphMX6*
**MY6996**	*h- leu1-32 ura4D-18 his3-D1 rhp6::KanMX6* (gifted by NBRP, Japan; originally made by Dr T. Toda)
**SP660**	*h- ade6-704 leu1-32 ura4D-18 his3-D1 chk1-HA*
**SP891**	*h- ade6-704 leu1-32 ura4D-18 his3-D1 chk1-HA wdr70::ura4+*
**SP925**	*h+ leu1-32 ura4D-18 his3-D1 chk1-HA cdc25-22*
**SP927**	*h+ leu1-32 ura4D-18 his3-D1 chk1-HA cdc25-22 wdr70::ura4+*
**SP865**	*h+ leu1-32 ura4D-18 his3-D1 ddb1::kanMX6 spd1::hphMX6*
**SP1099**	*h+ leu1-32 ura4D-18 his3-D1 rhp6::KanMX6 spd1::hphMX6*
**Roz 8**	*h- ade6-704 leu1-32 ura4D-18 his3-D1 ubp8::NatMX6* (gifted by Prof Alan Lehmann)
**SP1119**	*h+ ade6-704 leu1-32 ura4D-18 his3-D1 ubp8::NatMX6 wdr70::ura4+*
**SP1130**	*h+ ade6-704 leu1-32 ura4D-18 his3-D1 ubp8::NatMX6 rhp6::kanMX6*
**YY4138**	*h+ leu1-32 ura4-D18 his3-D1 arg3::HOsite(kanMX6) ars1::nmt-HO endonuclease:ampR:his3+:ars1* (gifted by Dr Paul Russell)
**YY4140**	*h- leu1-32 ura4-D18 his3-D1 rad11-TAP:kanMX6 arg3::HOsite(kanMX6) ars1::nmt-HO endonuclease:ampR:his3+:ars1*(gifted by Dr Paul Russell)
**YY4141**	*h-leu1-32 ura4-D18 his3-D1 rad11-TAP:kanMX6 mre11::natMX6 arg3::HOsite(kanMX6) ars1::nmt-HO endonuclease:ampR:his3+:ars1*(gifted by Dr Paul Russell)
**SP1026**	*h+ leu1-32 ura4-D18 his3-D1 spd1::hphMX6 arg3::HOsite(kanMX6) ars1::nmt-HO endonuclease:ampR:his3+:ars1*

**SP1027**	*h+ leu1-32 ura4-D18 his3-D1 spd1::hphMX6 wdr70::ura arg3::HOsite(kanMX6) ars1::nmt-HO endonuclease:ampR:his3+:ars1*
**SP1108**	*h+ leu1-32 ura4-D18 his3-D1 wdr70::ura arg3::HOsite(kanMX6) ars1::nmt-HO endonuclease:ampR:his3+:ars1*
**SP1104**	*h+ leu1-32 ura4-D18 his3-D1 spd1::hphMX6 htb1-K119R-3FLAG::kanMX6 arg3::HOsite(kanMX6) ars1::nmt-HO endonuclease:ampR:his3+:ars1*
**SP1105**	*h+ leu1-32 ura4-D18 his3-D1 htb1-K119R-3FLAG::kanMX6 arg3::HOsite(kanMX6) ars1::nmt-HO endonuclease:ampR:his3+:ars1*
**SP1162**	*h+ leu1-32 ura4-D18 his3-D1 spd1::hphMX6 ddb1::kanMX6 arg3::HOsite(kanMX6) ars1::nmt-HO endonuclease:ampR:his3+:ars1*
**SP1173**	*h+ leu1-32 ura4-D18 his3-D1 spd1::hphMX6 wdr70::kanMX6 rqh1::ura4+ arg3::HOsite(kanMX6) ars1::nmt-HO endonuclease:ampR:his3+:ars1*
**SP1174**	*h+ leu1-32 ura4-D18 his3-D1 spd1::hphMX6 rqh1::ura4+ arg3::HOsite(kanMX6) ars1::nmt-HO endonuclease:ampR:his3+:ars1*
**SP1176**	*h+ leu1-32 ura4-D18 his3-D1 spd1::hphMX6 wdr70::kanMX6 exo1::ura4+ arg3::HOsite(kanMX6) ars1::nmt-HO endonuclease:ampR:his3+:ars1*
**SP1160**	*h+ leu1-32 ura4-D18 his3-D1 spd1::hphMX6 exo1::ura4+ arg3::HOsite(kanMX6) ars1::nmt-HO endonuclease:ampR:his3+:ars1*
**SP1107**	*h+ leu1-32 ura4-D18 his3-D1 spd1::hphMX6 wdr70::ura4+ rad11-TAP:kanMX6 arg3::HOsite(kanMX6) ars1::nmt-HO endonuclease:ampR:his3+:ars1*
**SP1115**	*h+ leu1-32 ura4-D18 his3-D1 spd1::hphMX6 rhp6-TAP::kanMX6 arg3::HOsite(kanMX6) ars1::nmt-HO endonuclease:ampR:his3+:ars1*
**SP1116**	*h+ leu1-32 ura4-D18 his3-D1 spd1::hphMX6 wdr70::ura4+ rhp6-TAP::kanMX6 arg3::HOsite(kanMX6) ars1::nmt-HO endonuclease:ampR:his3+:ars1*
**SP1117**	*h+ leu1-32 ura4-D18 his3-D1 spd1::hphMX6 wdr70-TAP::kanMX6 arg3::HOsite(kanMX6) ars1::nmt-HO endonuclease:ampR:his3+:ars1*
**SP1118**	*h+ leu1-32 ura4-D18 his3-D1 spd1::hphMX6 htb1-K119R-3FLAG::kanMX6 wdr70-TAP::kanMX6 arg3::HOsite(kanMX6) ars1::nmt-HO endonuclease:ampR:his3+:ars1*
**SP1151**	*h+ leu1-32 ura4-D18 his3-D1 spd1::hphMX6 ctp1-TAP::kanMX6 arg3::HOsite(kanMX6) ars1::nmt-HO endonuclease:ampR:his3+:ars1*
**SP1152**	*h+ leu1-32 ura4-D18 his3-D1 spd1::hphMX6 wdr70::ura4+ ctp1-TAP::kanMX6 arg3::HOsite(kanMX6) ars1::nmt-HO endonuclease:ampR:his3+:ars1*
**SP1157**	*h+ leu1-32 ura4-D18 his3-D1 spd1::hphMX6 exo1-TAP::kanMX6 arg3::HOsite(kanMX6) ars1::nmt-HO endonuclease:ampR:his3+:ars1*
**SP1154**	*h+ leu1-32 ura4-D18 his3-D1 spd1::hphMX6 wdr70::ura4+ exo1-TAP::kanMX6 arg3::HOsite(kanMX6) ars1::nmt-HO endonuclease:ampR:his3+:ars1*
**SP1158**	*h+ leu1-32 ura4-D18 his3-D1 spd1::hphMX6 wdr70::ura4+ ddb1-TAP::kanMX6 arg3::HOsite(kanMX6) ars1::nmt-HO endonuclease:ampR:his3+:ars1*
**SP1183**	*h+ leu1-32 ura4-D18 his3-D1 exo1::ura4+ wdr70-TAP::kanMX6 arg3::HOsite(kanMX6) ars1::nmt-HO endonuclease:ampR:his3+:ars1*
**SP1185**	*h+ leu1-32 ura4-D18 his3-D1 spd1::hphMX6 rqh1::ura4+ wdr70-TAP::kanMX6 arg3::HOsite(kanMX6) ars1::nmt-HO endonuclease:ampR:his3+:ars1*
**SP1193**	*h+ leu1-32 ura4-D18 his3-D1 spd1::hphMX6 ctp1::kanMX6 wdr70-TAP::kanMX6 arg3::HOsite(kanMX6) ars1::nmt-HO endonuclease:ampR:his3+:ars1*
**SP1187**	*h+ leu1-32 ura4-D18 his3-D1 spd1::hphMX6 rqh1-TAP::kanMX6 arg3::HOsite(kanMX6) ars1::nmt-HO endonuclease:ampR:his3+:ars1*
**SP1194**	*h+ leu1-32 ura4-D18 his3-D1 spd1::hphMX6 wdr70::ura4+ rqh1-TAP::kanMX6 arg3::HOsite(kanMX6) ars1::nmt-HO endonuclease:ampR:his3+:ars1*
**SP1192**	*h+ leu1-32 ura4-D18 his3-D1 spd1::hphMX6 wdr70::ura4+ htb1-K119R-3FLAG::kanMX6 exo1-TAP::kanMX6 arg3::HOsite(kanMX6) ars1::nmt-HO endonuclease:ampR:his3+:ars1*
**SP1253**	*h+ leu1-32 ura4-D18 his3-D1 spd1::hphMX6 rhp6-TAP::kanMX6 exo1::ura4+ arg3::HOsite(kanMX6) ars1::nmt-HO endonuclease:ampR:his3+:ars1*
**SP1226**	*h+ leu1-32 ura4-D18 his3-D1 spd1::hphMX6 rhp6-TAP::kanMX6 rqh1:ura4+ arg3::HOsite(kanMX6) ars1::nmt-HO endonuclease:ampR:his3+:ars1*
**SP1234**	*h+ leu1-32 ura4-D18 his3-D1 spd1::hphMX6 rhp6-TAP::kanMX6 ctp1:ura4+ arg3::HOsite(kanMX6) ars1::nmt-HO endonuclease:ampR:his3+:ars1*
**SP1222**	*h+ ura4-D18 leu1-32 his3-D1 set9::NatMX6*
**SP1308**	*h+ leu1-32 ura4-D18 his3-D1 spd1::hphMX6 mre11-TAP::kanMX6 arg3::HOsite(kanMX6) ars1::nmt-HO endonuclease:ampR:his3+:ars1*

**SP1310**	*h+ leu1-32 ura4-D18 his3-D1 spd1::hphMX6 mre11-TAP::kanMX6 wdr70::ura4+ arg3::HOsite(kanMX6) ars1::nmt-HO endonuclease:ampR:his3+:ars1*
**SP1228**	*h+ leu1-32 ura4-D18 his3-D1 spd1::hphMX6 crb2-TAP::kanMX6 arg3::HOsite(kanMX6) ars1::nmt-HO endonuclease:ampR:his3+:ars1*
**SP1230**	*h+ leu1-32 ura4-D18 his3-D1 spd1::hphMX6 crb2-TAP::kanMX6 wdr70::ura4+ arg3::HOsite(kanMX6) ars1::nmt-HO endonuclease:ampR:his3+:ars1*
**SP1236**	*h+ leu1-32 ura4-D18 his3-D1 spd1::hphMX6 set9::NatMX6 arg3::HOsite(kanMX6) ars1::nmt-HO endonuclease:ampR:his3+:ars1*
**SP1237**	*h+ leu1-32 ura4-D18 his3-D1 spd1::hphMX6 set9::NatMX6 wdr70::ura4+ arg3::HOsite(kanMX6) ars1::nmt-HO endonuclease:ampR:his3+:ars1*
**SP1257**	*h+ leu1-32 ura4-D18 his3-D1 spd1::hphMX6 crb2-TAP::kanMX6 set9::NatMX6 arg3::HOsite(kanMX6) ars1::nmt-HO endonuclease:ampR:his3+:ars1*
**SP1256**	*h+ leu1-32 ura4-D18 his3-D1 spd1::hphMX6 crb2-TAP::kanMX6 set9::NatMX6 wdr70::ura4+ arg3::HOsite(kanMX6) ars1::nmt-HO endonuclease:ampR:his3+:ars1*
**Dy3766**	*h+ leu1-32 ura4-D18 ade6-L469/pUC8/his3/ade6-M375 his3-D1 arg3-D1*
**SP586**	*h+ leu1-32 ura4-D18 wdr70::ura4+ ade6-L469/pUC8/his3/ade6-M375 his3-D1 arg3-D1*
**SP1297**	*h+ leu1-32 ura4-D18 his3-D1 spd1::hphMX6 crb2-F400A exo1-TAP::kanMX6 arg3::HOsite(kanMX6) ars1::nmt-HO endonuclease:ampR:his3+:ars1*
**SP1306**	*h+ leu1-32 ura4-D18 his3-D1 spd1::hphMX6 crb2-F400A wdr70::ura4+ exo1-TAP::kanMX6 arg3::HOsite(kanMX6) ars1::nmt-HO endonuclease:ampR:his3+:ars1*

Supplementary Table 3. List of antibodies used in this study

Antibody	Source	Dilution
Rabbit anti-Myc	Millipore (05-724)	1:500
Rat anti-HA	Roche (11867423001)	1:500
Rabbit anti-TAP	sigma (p3775)	1:50000
Rabbit anti Flag	Huaan biological (Hangzhou, China)	1:1000
Mouse anti-Tubulin	sigma (T6074)	1:10000
Mouse anti-GFP	Roche (1814460001)	1:500
Rabbit anti-phospho Ser27 H1	Millipore (09-836)	1:200
Mouse anti-mono-ubiquitinated H2A	Millipore (05-678)	1:200
Rabbit anti-mono-ubiquitinated H2AX	Millipore (AB10029)	1:200
Mouse anti-mono-ubiquitinated H2B	Millipore (05-1312)	1:200
Mouse anti-dimethyl H4K20	upstate (07-031)	1:200
Mouse anti-trimethyl H4K20	Millipore (07-463)	1:200
Rabbit anti-H2B	Chemicon (ab1623)	1:20000
Mouse anti-H3	Millipore (05-1341)	1:10000
Mouse anti-dimethyl H3K4	Millipore (05-1338)	1:200
Mouse anti-trimethyl H3K4	Millipore (05-1339)	1:200
Rabbit anti-dimethyl H3K9	Upstate (07-212)	1:200
Mouse anti-trimethyl H3K9	Millipore (05-1250)	1:200
Mouse anti-trimethyl H3K27	Millipore (ABE44)	1:200
Rabbit anti-dimethyl H3K79	Millipore (04-835)	1:200
Rabbit anti-phospho Ser28 H3	Millipore (07-145)	1:200
Rabbit anti-acetylated Lys56 H3	cell signaling (#4243P)	1:200
Rabbit anti-H4	Millipore (07-108)	1:5000
Rabbit anti-53BP1	Bethyl, A300-272A	1:500
Rabbit anti-phosphor-Serine 33, Rpa32	NOVUS, NB100-544	1:500
Mouse anti-Rpa32	Santa Cruz, sc-271578	1:100
Rabbit anti-WDR70	Bethyl, A301-871A-1	1:500
Rabbit anti-Rad51	Proteintech, 14961-1-AP	1:1000
HRP-conjugated anti-mouse IgG	DAKO (P0260)	1:2000
HRP-conjugated anti-rabbit IgG	DAKO (P0448)	1:3000
FITC- conjugated anti-mouse IgG	Sigma (F0257)	1:200

Supplementary Table 4. List of plasmids used in this study

Plasmids	Source
Rep41-EGFP-Wdr70	This study
Rep41-EGFP-Wdr70-WD	This study
pLVX-G-sh53BP1	Purchased from Genechem

Supplementary Table 5. List of primers used in this study

	Sequence	Application
PRIMER 1	ATACTCCTAAAATTGATAGTTTATTC	Crb2-F400A Sequencing
PRIMER 2	GCTTGATTTTTCCTAATCTTGTG	Crb2-F400A Sequencing
PRIMER 3	AAAGTAGGCCGGCATCGAAAATTAAACATCCCCAAACC TATTCCAAATGGTTTTTGGGAAAGTGATTTTGTAGATCG G ATC CCC GGG TTA ATT A	Ctp1 tagging, forward
PRIMER 4	CTATGTACACGGTTGGCGTATCAATTTATTTAGACAAGTT GTCATTGGATGAAGTTTCACAATCATTATTTAGTTGAATT CGAGCTCGTTTAAAC	Ctp1 tagging, reverse
PRIMER 5	AAAAGACTCTCGAAAAACTTCTAAAAGTGCCAACACAT CATTCATACATCCCATGGTTAAGCAAAATTATCGT CGGATCCCCGGGTTAATTAA	Rqh1 tagging, forward
PRIMER 6	TTTCTAACGTATTATAGACAATGTTTAAATGAACGCACAT GTACAATAAACGAACCATAAATTAACAGGAATAAAGAAT TCGAGCTCGTTTAAAC	Rqh1 tagging, reverse
PRIMER 7	CTAGTCCCTCAACCCCAATATCTATGAATCCTAGACCTAA AGGAATCCTATCACTTCAGCAATATAAATTTCGTCGGATC CCCGGGTTAATTAA	Exo1 tagging, forward
PRIMER 8	CATAAATCGTAATTACTTAAAACCGCACCGTTCATAAAG TAATTAACCCAGTTGGTGATATAATGAGAAAGGTG GAATTCGAGCTCGTTTAAAC	Exo1 tagging, reverse
PRIMER 9	GCTCAGCTGCATCGTGAAAACAAGAAGGAGTATGTGCG TCGAGTACGAAAAACTGTAGAAGACTCCTGGGAAAGTC GGATCCCCGGGTTAATTAA	Rhp6 tagging, forward
PRIMER 10	CGTAGATGTAAACAAAAAAATCTTAATGATTTACTATAGT TACAATTTTGGCAACTTTGAAGCACTTAAATAGGAATTC GAGCTCGTTTAAAC	Rhp6 tagging, reverse
PRIMER 11	CGTCCTTCCGATGTTGCTTTAACGCATACTC	HO TOTAL
PRIMER 12	AGACGTATTTGAGTGATAGTGCTCGCTGC	HO TATOL
PRIMER 13	TGTTGCGGAAAGCTGAAAGGTACCTG	HO site 35bp amplicon, forward
PRIMER 14	CTCGCAGTCTGAGAGAGAACTAGATATCGG	HO site 35bp amplicon, reverse
PRIMER 15	CATAAGGTTTGCATACACCGTTGGGTAGG	HO site 3kb amplicon, forward
PRIMER 16	CGGAAAGAACTTGATTGGATTGATTAACACTCATCC	HO site 3kb amplicon, reverse
PRIMER 17	CGTACTAGCTTGTTTGCAACTGAACTCTAGTG	HO site 0.2kb amplicon, forward
PRIMER 18	CCGATATCTAGTTCTCTCTCAGACTGCGAG	HO site0.2kb amplicon, reverse
PRIMER 19	TAGCACCGGCTCGTCTATTT	HO site 5.4kb amplicon, forward
PRIMER 20	AAGCAATGGGACTTCAATCG	HO site 5.4kb amplicon, reverse
PRIMER 21	TCAAAGCTGCGAAACAACAC	HO site 9kb amplicon, forward
PRIMER 22	TCGGTGCAGACGATCAATAA	HO site 9kb amplicon, reverse
PRIMER 23	ATACGACTCACTATAGGGCGAATTGGGTAC	HO uncut
PRIMER 24	GTCAAGGAGGGTATTCTGGGCCTCCATG	HO uncut
PRIMER 25	GCCCTTCGTGTTGCGGCCTTGCGAAATTCTAA	W325A D326A site directed mutagenesis

PRIMER 26	TTAGAATTTCGCAAGGCCGCAACACGAAGGGC	W325A D326A site directed mutagenesis
PRIMER 27	CCAA CTCGAG ATGGATGGCA CAAATAATAC	Wdr70/Rep41-EGFP, forward
PRIMER 28	CCAA AGATCT TCTTTTTTGCTTTTTTGAAGGGTTTCC	Wdr70/Rep41-EGFP, reverse
PRIMER 29	AGTTAAATGGCTATTTGAAAAAAATAATAGGGAAACAAT ATATCAGCCTCTAAAATTTTACAATATCCAGATTTTCGTA A AATTAACCCTCACTAAAGG	Wdr70 deletion (Baler system)
PRIMER 30	GTTGATTTTCATTTTTTTCAACTTTAGTTTTCATCTTTTTT GCTTTTTTGAAGGGTTTCCTTCTTCAGAACTAATATCTCC TAATACGACTCACTATAG	Wdr70 deletion (Baler system)
PRIMER 31	AGCCAACTCCCATTTATCAAGGTGTTACAGAAGGAGATA TTAGTTCTGAAGAAGGAAACCCTTCAAAAAAGCAAAA AAGACGG ATC CCC GGG TTA ATT AA	Wdr70 tagging, forward
PRIMER 32	GAGCTTAAACTTTTTTGTCATTATGTAAAGCTGAATAAC ATCGCTAAATATTAATAAGTCCATCAATCTATCTTCCCAC A GAATTCGAGCTCGTTTAAAC	Wdr70 tagging, reverse
PRIMER 33	CTTCTATTTTACGACCCATCCAGCACCACAGAAGCGCAG TACTTGGATAACGAGGATGACGAAATTTTAGATGAT CGG ATC CCC GGG TTA ATT AA	Mre11 tagging, forward
PRIMER 34	AGAACCTACAACAATGGGCTTAACATTACAGTATCCAAA TATAGGTACACTTAAACAAAAATTTGATAAGATCAA GAATTCGAGCTCGTTTAAAC	Mre11 tagging, reverse
Primer 35	ATCATGGCCGACAAGCAGAAGAACG	Normalization for I-SceI system, forward
Primer 36	CGGCGGCGGTCACGAACTCC	Normalization for I-SceI system, reverse
Primer 37	TGACCACCCTGACCTACG	HR/SSA repair, forward
Primer 38	CACCTTGATGCCGTTCTTCTGC	repair, reverse
Primer 39	TCGGAGCAAGCTTGATTTAGGTGA	NHEJ repair, forward
Primer 40	AAATTAGCCGGCACCTGTAATCCC	WDR70 Exon 1 sequencing, forward
Primer 41	GTTGGTTCCAACGGATAACGCAGA	WDR70 Exon 1 sequencing, reverse
